# Mitochondrial dysfunction is a key pathological driver of early stage Parkinson’s

**DOI:** 10.1186/s40478-022-01424-6

**Published:** 2022-09-08

**Authors:** Christina E. Toomey, Wendy E. Heywood, James R. Evans, Joanne Lachica, Sarah N. Pressey, Sandrine C. Foti, Mesfer Al Shahrani, Karishma D’Sa, Iain P. Hargreaves, Simon Heales, Michael Orford, Claire Troakes, Johannes Attems, Ellen Gelpi, Miklos Palkovits, Tammaryn Lashley, Steve M. Gentleman, Tamas Revesz, Kevin Mills, Sonia Gandhi

**Affiliations:** 1 Queen Square Brain Bank for Neurological Disorders, UCL Queen Square Institute of Neurology, London, UK; 2 Department of Clinical and Movement Neurosciences, UCL Queen Square Institute of Neurology, London, UK; 3 The Francis Crick Institute, London, UK; 4 Translational Mass Spectrometry Research Group, Genetic & Genomic Medicine, Institute of Child Health, UCL, London, UK; 5 Department of Neurodegenerative Disease, UCL Queen Square Institute of Neurology, London, UK; 6 National Hospital for Neurology and Neurosurgery & Neurometabolic Unit, UCL Great Ormond Street Institute of Child Health, London, UK; 7 College of Applied Medical Sciences, King Khalid University, Abha, Saudi Arabia; 8 London Neurodegenerative Diseases Brain Bank, Institute of Psychiatry, Psychology & Neuroscience, King’s College London, London, UK; 9 Newcastle Brain Tissue Resource, Institute of Neuroscience and Newcastle University Institute for Ageing, Newcastle upon Tyne, UK; 10 Neurological Tissue Bank, University of Barcelona, Barcelona, Spain; 11 Division of Neuropathology and Neurochemistry, Department of Neurology, Medical University of Vienna, Vienna, Austria; 12 Human Brain Tissue Bank, Budapest, Semmelweis University, Budapest, Hungary; 13 Department of Brain Sciences, Imperial College London, London, UK

**Keywords:** Parkinson’s, Mitochondria, Brain, Progression, Neurodegeneration, Proteomics

## Abstract

**Background:**

The molecular drivers of early sporadic Parkinson’s disease (PD) remain unclear, and the presence of widespread end stage pathology in late disease masks the distinction between primary or causal disease-specific events and late secondary consequences in stressed or dying cells. However, early and mid-stage Parkinson’s brains (Braak stages 3 and 4) exhibit alpha-synuclein inclusions and neuronal loss along a regional gradient of severity, from unaffected-mild-moderate-severe. Here, we exploited this spatial pathological gradient to investigate the molecular drivers of sporadic PD.

**Methods:**

We combined high precision tissue sampling with unbiased large-scale profiling of protein expression across 9 brain regions in Braak stage 3 and 4 PD brains, and controls, and verified these results using targeted proteomic and functional analyses.

**Results:**

We demonstrate that the spatio-temporal pathology gradient in early-mid PD brains is mirrored by a biochemical gradient of a changing proteome. Importantly, we identify two key events that occur early in the disease, prior to the occurrence of alpha-synuclein inclusions and neuronal loss: (i) a metabolic switch in the utilisation of energy substrates and energy production in the brain, and (ii) perturbation of the mitochondrial redox state. These changes may contribute to the regional vulnerability of developing alpha-synuclein pathology. Later in the disease, mitochondrial function is affected more severely, whilst mitochondrial metabolism, fatty acid oxidation, and mitochondrial respiration are affected across all brain regions.

**Conclusions:**

Our study provides an in-depth regional profile of the proteome at different stages of PD, and highlights that mitochondrial dysfunction is detectable prior to neuronal loss, and alpha-synuclein fibril deposition, suggesting that mitochondrial dysfunction is one of the key drivers of early disease.

**Supplementary Information:**

The online version contains supplementary material available at 10.1186/s40478-022-01424-6.

## Background

Neurodegenerative diseases are progressive, devastating and incurable, and are becoming increasingly prevalent in our ageing population [1]. Parkinson’s disease (PD) is the most common neurodegenerative movement disorder, affecting 1–2% of people aged over 65, with an estimated > 6.1 million people affected worldwide [2]. PD presents with clinical symptoms at an advanced stage of disease, when intraneuronal inclusions known as Lewy bodies (LB), composed primarily of insoluble aggregates of the protein alpha-synuclein, mitochondria, and lipid membranes, and significant neuronal loss are evident [3, 4]. Based on a cross-sectional study of post-mortem PD brain, Braak proposed that nigral and extranigral pathology do not occur simultaneously, but rather, LB pathology develops sequentially across the brain in anatomically connected regions in a caudal-rostral manner [5]. Whilst this progression of pathology may be controversial, several studies have confirmed that PD brains demonstrate alpha-synuclein positive lesions and neuronal loss in medullary, pontine and midbrain nuclei, with further pathology in the nucleus basalis of Meynert (90–98%), limbic cortex (50–60%), cingulate cortex (32–46%), frontal cortex (29–31%), and amygdala (25%), illustrating that the susceptible regions in PD and their interconnectivity remains consistent across studies [6, 7].

Genetic studies of familial and sporadic PD strongly suggest that altered expression, concentration, or mutant forms of alpha-synuclein are central to the pathogenesis of PD [8–11]. Furthermore, experimental and cellular studies suggest that alpha-synuclein may spread from neuron to neuron in a prion-like manner, inducing seeding and toxicity in neighbouring cells [12, 13]. Despite this, the mechanisms that cause toxicity, and lead to the pathological spread of disease throughout the brain, are still largely unknown. As no cell or animal model fully recapitulates the spatio-temporal spread of protein aggregates and neuronal loss of PD, such mechanisms can only be studied in PD patient brain tissue. Although, post-mortem brain is a powerful source of understanding the human disease, it is unable to distinguish disease-specific signals from the non-disease related ‘noise’ from stressed or dying cells in regions with advanced stage pathology. In regions with end-stage disease, there are large amounts of cell death, protein aggregation, chronic inflammation, oxidative stress and neurotoxicity and therefore many analyses of end stage tissue predominantly capture these pathways. Distinguishing whether these processes are causal, specific to Parkinson’s pathogenesis, and occur from the start of the condition, or whether they result from secondary consequences of dead or dying neurons is important in the search for the early events driving disease. Unbiased large-scale profiling of protein expression across thousands of proteins in specific brain regions, with sequential levels of pathology, therefore, has the potential to distinguish these signals, and uncover the mechanisms underlying brain region vulnerability and spread of pathology. However, proteomic studies investigating PD often focus on minimal brain regions (predominantly substantia nigra or frontal cortex) and/or focus on late stage disease where extensive neuronal loss can be observed [14–20]. Dixet et al [21]. reviewed the need for additional brain regions to be studied simultaneously to understand how protein expression changes with disease progression.

In this study, we address this gap in knowledge through the comprehensive investigation of the earliest changes in cerebral protein expression in PD. To achieve this, we selected cases with LB pathology corresponding to Braak stage 3 or 4, in which pathology is restricted to the brainstem, striatum and antero-medial temporal cortex, and not found in frontal and parietal cortices. We sampled across the 9 brain regions affected in PD, within any individual case, to capture the disease signature at mild, moderate, and severe disease stages, according to Braak staging. This approach enabled us to identify disease-specific convergent mechanisms underlying cell specific vulnerability. We utilised early-mid stage PD tissue to identify pathways common across disease regions, including those with minimal pathology, thus suggesting their involvement early in disease pathogenesis. We were able to identify proteins that exhibit altered expression prior to the appearance of pathology (unaffected regions), highlighting pathways that are dysregulated at the earliest stages of PD, and therefore likely driving the disorder. Together, the use of anatomically driven proteomic analyses, and the validation of targets through targeted proteomic and functional investigation, has identified critical disease mechanisms likely underpinning the pathogenesis of PD.

## Materials and methods

### Tissue

Post-mortem brain tissue was obtained from the Neurological Tissue Bank, IDIBAPS-HC-Biobanc, Barcelona; Human Brain Tissue Bank, Budapest; UK Parkinson’s Disease Society Tissue Bank, Imperial College London; the London Neurodegenerative Diseases Brain Bank, Institute of Psychiatry, King’s College, London; Netherlands Brain Bank, Amsterdam; and the Newcastle Brain Tissue Resource. Informed consent was given in all cases. Cases and controls were matched as close as possible for age and sex and all had a post-mortem delay of less than 20 h. Details of cases used are listed in Table 1. Control subjects did not have a diagnosis of neurological disease in life and at post-mortem displayed age-related changes only. All cases were assessed for alpha-synuclein pathology and rated according to Braak staging [5]. PD cases were categorised as either Braak stage 3 or 4 (early) or Braak stage 6 (late). Ethical approval for the study was obtained from the Local Research Ethics Committee of the National Hospital for Neurology and Neurosurgery.

**Table 1:**
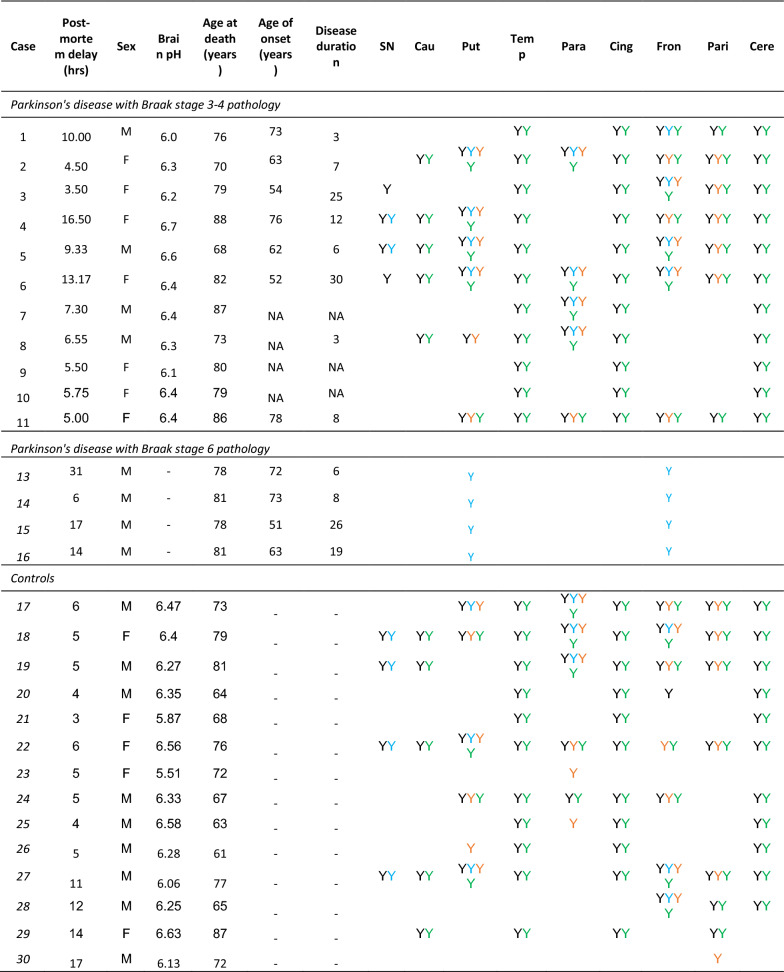
Case demographics table

Y indicates which cases were used from each region for first pooled proteomic run (black), second individual sample proteomic run (blue), functional mitochondrial assays (orange), and multiple reaction monitoring (green)

### Dissection of regions of interest

Samples were taken where available (outlined in Table 1) from the Braak staging areas; substantia nigra, caudate nucleus, putamen, cingulate cortex, parahippocampal cortex, temporal neocortex, frontal cortex, parietal cortex, and as a control region, the cerebellum. To ensure samples contained only the region of interest and were not contaminated with white matter or surrounding nuclei, tissue was sampled from the appropriate frozen tissue blocks using a punch technique [22]. Accordingly, approximately 300–500 μm thick sections were cut in a cryostat at − 7 °C and for the removal of specific cortical regions and nuclei with the help of special hollow needles, ranging from 3 to 5 mm in diameter. Between 20 and 60 μg tissue was collected per region and samples were subsequently stored at − 80 °C until required [23].

### Tissue preparation

Samples were prepared in two fractions a ‘soluble fraction’ and an ‘insoluble fraction’. Tissue was homogenised on ice in 500 μl of 50 mM Ambic buffer, 2% ASB-14 using the TissueRuptor mechanical homogeniser (Qiagen). After sonication, protein concentration was assessed using the bicinchoninic acid protein assay kit (Thermo Scientific). For initial LC–MS/MS analysis, a pool was created of equal amounts of PD cases or controls for each region to make a total pool of 1 mg of protein. At the same time, 300 μg of protein for each individual sample was collected for individual analyses. Pooled and individual samples then underwent acetone precipitation and the pellet fractions were treated with 70% formic acid as detailed previously [24].

### In-solution digestion

Pooled freeze dried brain tissue samples were subjected to in-solution digestion as described previously [25, 26]. Briefly samples were resuspended and reduced using dithioerythritol then subsequently carboamindomethylated using iodoacetamide and digested using 1 μg of sequence grade Lys-C and trypsin (Promega).

### High pH fractionation

Pooled peptide samples were fractionated at high pH prior to proteomic analysis to provide a greater depth of coverage of the proteome. 400 μl of 0.2% ammonia was added to 100 μl of the peptide solution, vortexed and spun at 13,000 g for 5 min. 50 mg Isolute C18 columns (Biotage) were primed with 1 ml of 50% acetonitrile containing 0.1% ammonia, followed by two 1 ml washes with 0.1% ammonia solution. The peptide mixture was next added to the column and allowed to flow-through under gravity. The initial breakthrough was collected, re-applied to the column and the subsequent breakthrough collected. 500 μl of 0.1% ammonia was used to wash the column and the peptides were subsequently eluted into 7 fractions with 500 μl of increasing concentrations of acetonitrile containing 0.1% ammonia; 5%, 8%, 10%, 15%, 25%, 100% and finally 100% methanol. All fractions were dried by centrifugal evaporation and then reconstituted in 3% acetonitrile, 0.1% trifluoroacetic acid. Prior to mass-spec analysis, peptides were spiked with 100fmol MassPREP™ Enolase (Waters UK).

### Label-free quantitative MS

The first proteomic analysis was performed using MS^e^ label free proteomics on a nanoAcquity nano-LC and QTOF Premier (Waters Corporation, Manchester, UK) as described previously [27] on pooled samples in 9 brain regions (substantia nigra, putamen, caudate nucleus, parahippocampal cortex, temporal cortex, cingulate cortex, frontal cortex, parietal cortex and cerebellum) in the first instance. Peptides were trapped, desalted, and separated as described previously [27].

The second proteomic analysis was performed on four Braak stage 3/4 cases and four controls as individual samples from the substantia nigra, putamen, parahippocampus and frontal cortex as previously described [28] using a nanoAquity coupled to a Synapt-G2-Si mass spectrometer with high definition ion mobility capability (Waters, UK) with online 2D fractionation. This enables deeper phenotyping than the first run using a high definition more sensitive mass spectrometer. Additionally, two Braak stage 6 samples were run from the putamen and frontal cortex. Samples were prepared and run using the same methods as above other than high pH fractionation procedures.

### Data analysis of brain samples analysed by LC–MS/MS

First proteomic analysis: ProteinLynx GlobalServer version 2.4 (Waters Corporation) was used to process all data acquired from the first run as described previously [27]. Protein identifications were obtained by searching UniProt human reference proteome canonical database (June 2016): 1 missed cleavage, 4% false discovery rate and fixed modifications of carboamidomethylation of cysteines and dynamic modifications of oxidation of methionine.

Second proteomic analysis: Data acquired using the Synapt G2 MS were analysed using Progenesis LC–MS (Nonlinear Dynamics Limited, Newcastle, UK) raw data was processed as described previously but with the Uniprot database (June 2019) [24]. Briefly peptides were searched as described above except with 1% false discovery rate. Protein data for identifications with a confidence score > 20 and more than one unique peptide were exported for further analysis.

### Bioinformatics

As a label-free approach was taken, all genes or proteins that met the threshold set (> twofold change in one region, and also changing in four other regions) were subject to gene ontology analysis using Webgestalt [29, 30] and DAVID (version 6.8, [31]). GOview was used to compare terms that were over-represented between regions or disease groups (http://www.webgestalt.org/2017/GOView/). Ingenuity Pathway Analysis software (Qiagen) was used to perform in depth canonical pathway analysis and determine biological functions altered in the datasets.

Multivariate analysis on the second data analysis on the Synapt G2 MS was performed using SIMCA v15 (Umetrics, Sweden). The mitochondrial sub proteome analysis was done using Mitominer to annotate all proteins in the dataset as to mitochondria association by GO [32]. Mitochondrial proteins were grouped according to main mitochondrial functions using GO Panther [33].

Analysis of the cell type-specific protein expression was performed in a number of available datasets: mouse brain proteomics generated by Sharma et al. [34], human brain transcriptomics and proteomics [35, 36] and mouse PD transcriptomics [37] to assess for enrichment of the significantly associated proteins/genes in particular brain cell types. Enrichment analysis was performed using Bioconductor R package GeneOverlap [38] that returns the Fisher’s exact test p-value and odds ratio as previously performed in Wingo et al, [39]. Multiple comparison test was performed using Benjamini & Hochberg method and only overlaps with FDR < 5% were considered significant.

### Targeted proteomic MRM LC–MS/MS assay

Potential candidates identified in the label free proteomics were selected for validation on the basis of  a change in expression over five of the nine brain regions with at least one region having a > twofold change. Secondly, proteins that had a > fivefold change in expression in any one region were added. Selected proteins were not included in the validation list eg. keratins, structural proteins, ribonucleoproteins. The assay was developed in the same way as shown in Heywood et al. [40]. A full list of peptides is given in Additional file 4: Table S1. Intact heavy labelled peptides were used as internal standards. Additionally, twenty nanograms of a generic yeast enolase whole protein standard (Sigma Aldrich, UK) was used as another level of internal standard. This was added to brain samples before they were processed from the individual protein extractions described above. Digested peptides were separated using a 50 mm C18^+^ UPLC column attached to a C18^+^ Vanguard and analysed using a Xevo TQ-S (Waters, Manchester, UK). UPLC and MS tune conditions were performed as previously described [40]. Samples were run in duplicate. Chromatograms were analyzed using Waters TargetLynx software. Peptides were standardized to an internal standard. Absolute levels were obtained from standard curves of custom synthesised peptides spiked into pooled brain digest.

### Mitochondrial enzyme activity assays

Punch dissections from putamen, frontal cortex and parietal cortex were also from 4–6 of the controls and 4–6 of the Braak stage 3/4 PD cases. These cases were age/gender (male (M) and female (F)) and post-mortem delay (PMD) matched (See Table 1). Each brain region was homogenised and mitochondrial respiratory chain complex I, II–III, IV, II and citrate synthase activities were assayed spectrophotometrically at 30 °C as described in the study by Hargreaves et al. [41].

### Immunohistochemistry

8 µm mounted FFPE tissue sections were incubated at 60 °C overnight. Sections were deparaffinised in Xylene and rehydrated in decreasing grades of alcohol. Slides were incubated in a methanol/hydrogen peroxide (0.3%) solution for 10 min to block endogenous peroxidase activity. Room temperature epitope retrieval and heat-induced epitope retrieval were utilised. Sections were first incubated in 98% formic acid for 10 min and then transferred to a boiling 0.1 M citrate buffer (pH 6.0) solution and pressure cooked at maximum pressure for 10 min. To block non-specific binding, sections were incubated in 10% non-fat milk in TBST for 30 min at room temperature. Mouse monoclonal alpha-synuclein [4D6] (abcam ab1903,1:10,000) primary antibody was diluted in TBST and sections were incubated in 200 µl of antibody solution for 1 h at room temperature. Sections were washed 3 times for 5 min in TBST and then were incubated for 30 min in 200 µl of biotinylated goat anti-mouse IgG secondary antibody (Vector Laboratories BA 9200, 1:200). Slides were washed as in the previous step and then incubated in pre-conjugated Strept(avidin)–Biotin Complex (ABC; DAKO) for signal amplification. Slides were then washed as in previous and were submerged in 5% 3,3ʹ-Diaminobenzidine (DAB) chromogen in TBST for 5 min then counterstained in Mayer’s haematoxylin (BDH). Finally, slides were dehydrated in increasing grades of alcohol (70, 90 and 100% IMS), cleared in xylene and mounted with DPX.

## Results

### Detection of proteins in human post-mortem brain

**Fig. 1:**
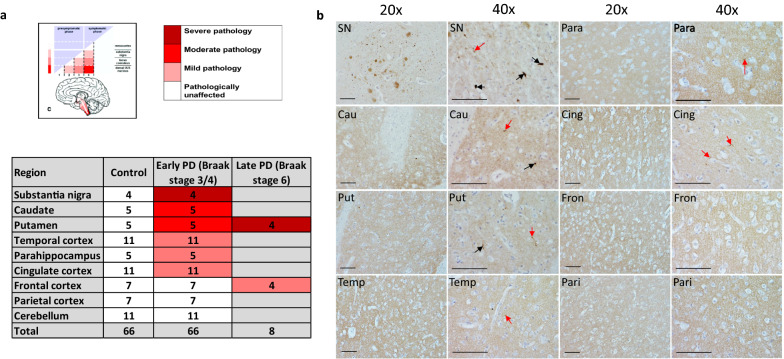
Case selection according to Braak staging system. **a** Diagram indicating the amount of Lewy body pathology in the regions used in the study according to Braak staging criteria. Severity of pathology at early-mid stage Braak 3/4 and late Braak stage 6 is highlighted by colours shown in reference to the key. Table indicates how many cases used for each region and disease group. **b** Panel of representative pathology images from a Braak stage 3/4 brain, highlighting the level of alpha-synuclein pathology in each region at this stage of disease at 20 × and 40 × magnification. Regions are labelled as follows: *SN* Substantia nigra; *Cau* Caudate; *Put* Putamen; *Temp* Temporal cortex; *Para* Parahippocampal gyrus; *Cing* Cingulate cortex; *Fron* Frontal cortex; *Pari* Parietal cortex. Black arrows show Lewy body presence and red arrows Lewy neurites. All scale bars represent 10 μm

Brains from 11 early-mid stage PD (Braak 3/4) cases were collected and specific regions were dissected according to tissue availability. Average age of death, average disease duration, and average post-mortem delay were calculated for all cases and matched to controls. The average post-mortem delay for cases was 7.92 h (range 3.5–16.5) and 9.21 h (range 3–23) for controls. Mean age of death for cases was 79 yrs (range 70–88) and 74 yrs (range 61–88) for controls. Average disease duration was 11.75 yrs (range 3–30). Mean pH in cases was 6.35 (range 6–6.67) and 6.30 in controls (range 5.51–6.63). Label free proteomic analysis was performed on 9 brain regions for PD and controls (pathological and clinical data outlined in Table 1). Brain regions consisted of substantia nigra, caudate nucleus, putamen, parahippocampal cortex, cingulate cortex, temporal cortex, frontal cortex, parietal cortex, and cerebellum. The degree of pathology was based on the Braak staging system and defined as ‘severe’ (substantia nigra), ‘moderate’ (caudate nucleus and putamen), ‘mild’ (parahippocampal cortex, cingulate cortex and temporal cortex), ‘unaffected’ (frontal cortex, parietal cortex and cerebellum). This is highlighted with representative pathology as shown in Fig. 1.

**Fig. 2:**
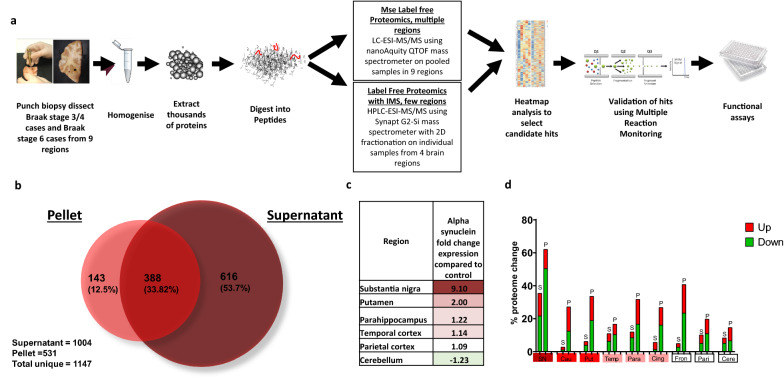
Workflow of methodology. **a** Flowchart highlighting methods used within the experiment. Tissue was micro-dissected per region, tissue homogenised and proteins were extracted and digested into peptides. Samples were proteome profile using mass spectrometry. Candidates were selected before confirmatory targeted Multiple Reaction Monitoring LCMS/MS analysis was completed. Functional assays were performed. **b** Venn diagram indicating how many proteins were detected uniquely in the supernatant and pellet fractions and how many overlapped. **c** Heat map of alpha-synuclein expression as fold change in Braak stage 3/4 compared to control indicated by colour. Red indicated upregulation and green downregulation compared to controls with deeper colour indicating a higher level of change. White indicates no change in expression compared to controls. **d** Overall change in the brain proteome for each region and condition expressed as a percentage of total proteome either upregulated (red) or downregulated (green) by more than 1.5 fold. S represents the supernatant fraction and P represents the pellet fraction. Graph created in GraphPad Prism v8

In the first proteomic analysis, samples were pooled by region (workflow shown in Fig. 2a). For each pool, the optimised fractionation and solubilisation methods enabled the detection of a total of 1147 proteins: consisting of 1004 supernatant proteins (soluble) and 531 pellet proteins (insoluble) (Fig. 2b). The level of insoluble alpha-synuclein, determined by mass spectrometry, followed the same spatial gradient as the LB pathology using standard immunohistochemical methods in the Braak staging system; with the substantia nigra showing the highest expression, and the levels decreasing in moderate, mild, and unaffected regions, with the lowest expression found in the cerebellum (Fig. 2c). This confirms the use of the staging system to assess pathology severity.

We calculated the % proteome change for each region in the soluble and insoluble fractions and found that the severely affected region (substantia nigra) was associated with the greatest proteome change, whilst moderate and mild regions had fewer changes in protein expression (Fig. 2d). Of note, the insoluble fraction had consistently more changes in protein levels than the soluble fraction across all regions.

### Bioinformatic analysis highlights mitochondrial dysfunction across the PD brain

**Fig. 3:**
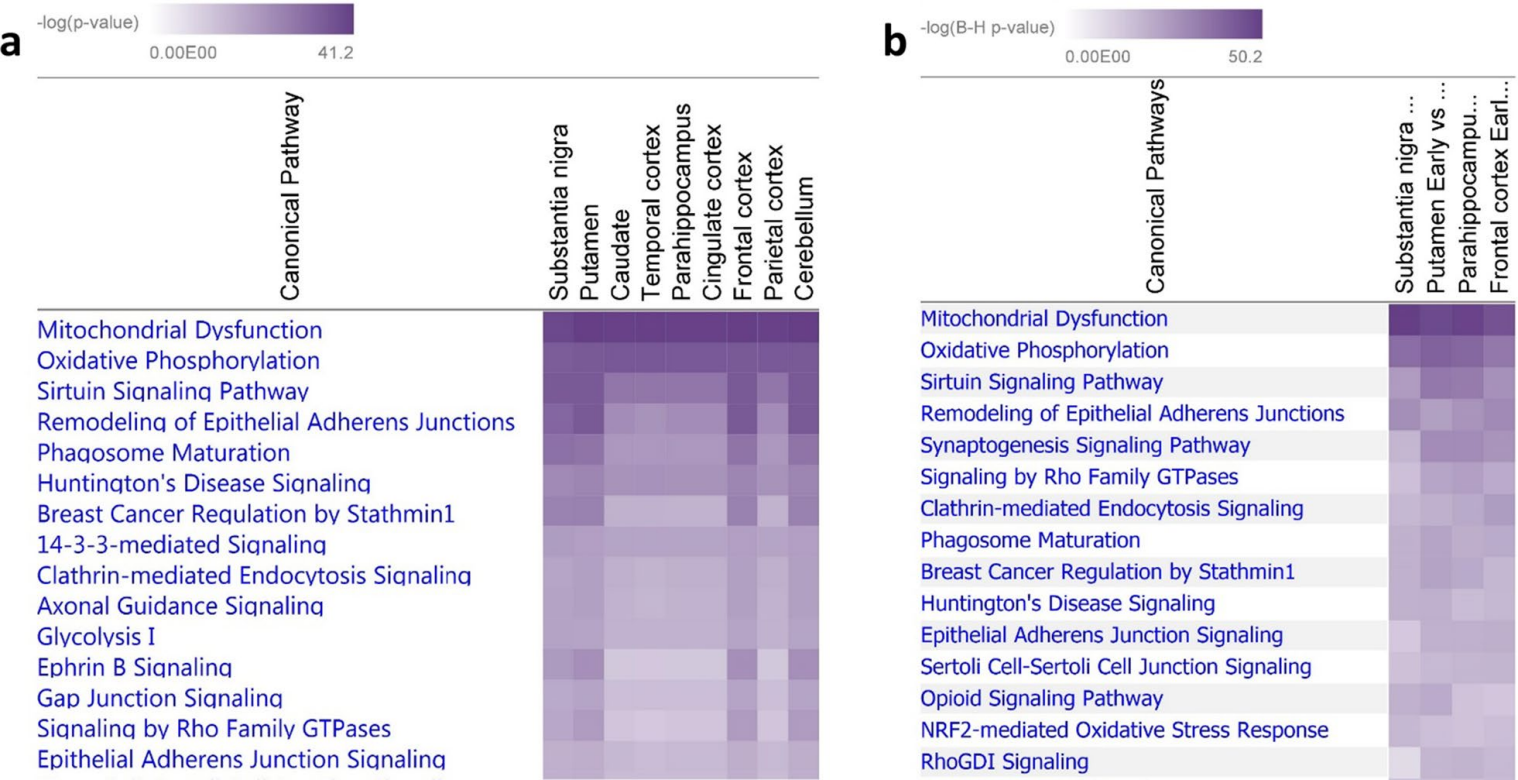
Pathway analysis of proteomics. Heatmap from IPA software showing the most significantly altered canonical pathways found in **a** the first mass spectrometry dataset and **b** the second mass spectrometry dataset with Mitochondrial dysfunction the top pathway significantly altered across all regions in Braak stage 3/4 compared to controls and in both datasets. The p-value is indicated by purple colour. The deeper the colour the lower the p-value

The fold change for all proteins in the PD brain (compared to controls) was analysed using IPA (see methods). The top canonical pathway, that was most significantly represented throughout the data across all regions, was mitochondrial dysfunction (see Fig. 3a). When observing the protein expression as fold change compared to control for the mitochondrial pathway, many of the proteins were upregulated (depicted in red), or down regulated (depicted in green; Additional file 1: Figure S1b).

Proteins that were changing in expression at > 1.5 fold (Table 2) were further assessed using Webgestalt [29, 30] and David v6.8 [31] to generate a cumulative list of gene ontology (GO) terms for each region (any duplicate terms across the software programs were excluded) in the sections Biological Process (BP), Molecular Function (MF) and Cellular Components (CC). In the substantia nigra (severe), there were a larger number of GO terms (276 BPs/135 MF/144 CCs) compared to mildly affected or unaffected regions (parietal region had 132 BPs/41MF/80 CCs), demonstrating that the degree of altered protein expression reflects the pathological severity of the region in PD brain (Additional file 1: Figure S1a). The full list of BPs/MF’s and CCs found across all regions are shown in Additional file 5: Tables S2. Additional file 6: Tables S3, Additional file 7: Tables S4 respectively.

**Table 2:** Differently expressed proteins across brain regions in early PD (Braak stage 3/4) compared to controls

	Upregulated	Downregulated	Total
Substantia nigra	159	301	***460***
Caudate	59	63	***122***
Putamen	69	96	***165***
Parahippocampus	72	100	***172***
Temporal cortex	45	64	***109***
Cingulate cortex	60	63	***123***
Frontal cortex	118	93	***211***
Parietal cortex	55	70	***125***
Cerebellum	58	70	***128***

The number of proteins that had an up- or down- regulation with > 1.5 fold expression according to each brain region tested

Next, we were interested in common pathways that are affected across multiple regions, and therefore ranked pathways according to their detection in different regions. 17 GO terms for BPs were affected in all regions; 9 GO terms for MF in all regions; 16 GO terms for CC in all regions. These common overlapping pathways in multiple regions (5 or more) included: translation, protein folding and assembly, metabolic and energy dependent processes (for BPs); activity of antioxidants, metabolism, and small molecule binding (for MFs). In the cellular compartment analysis, the top three compartments in all regions included extracellular space, cytoplasm, and mitochondria.

### Validation of proteome hits

**Fig. 4:**
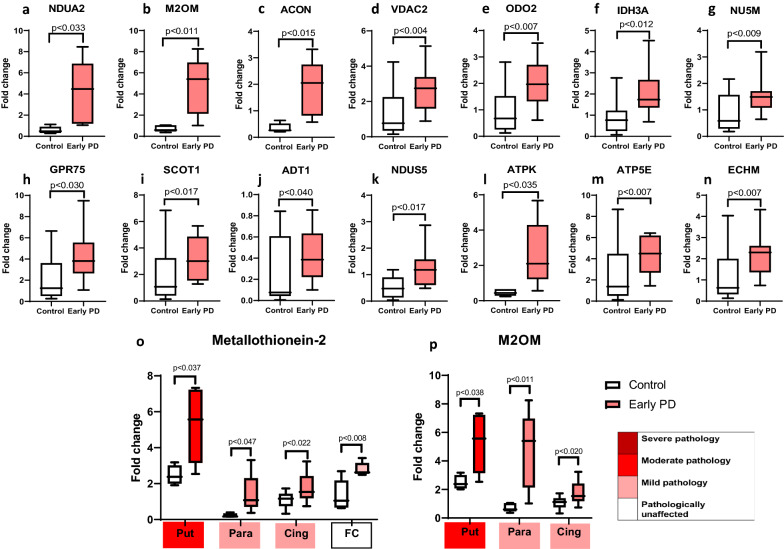
Validation of increased mitochondrial proteins in PD cases using multiple reaction monitoring LC–MS/MS. Graphs highlighting differences in fold change as a ratio against internal standard **a**–**n** for multiple mitochondrial proteins in the parahippocampus, **o** metallothionein-2 across the putamen, parahippocampus, cingulate cortex and frontal cortex, and **p** mitochondrial 2-oxoglutarate malate carrier across the putamen, parahippocampus and cingulate cortex. Regions are colour-coded for severity of pathology present at that stage of disease as shown in key. T-tests were done for each pairing and statistically significant results at *p* < 0.05 are highlighted on the graphs. Graphs and statistics completed with GraphPad Prism v8

To validate the hits generated from the list of differentially expressed candidate proteins (Table 3), we developed and performed targeted MRM LC–MS/MS analysis on 86 proteins across all early-mid PD cases (Braak stage 3/4), and controls, and brain regions as shown in Table 1. From this analysis we were able to quantify the expression fold changes of 73 proteins. Of these, 57 proteins were statistically significantly elevated in at least one region, 17 of which have mitochondrial localisation. Notably, the largest number of mitochondrial proteins (13) showed statistically significant changes in the parahippocampus, a region that exhibits only mild pathology. These are shown in Fig. 4, and include NDUA2 (4.12 fold increase, *p* = 0.033), M2OM (4.73 fold increase, *p* = 0.01), ACON (1.8 fold increase, *p* = 0.015), VDAC2 (2.64 fold increase, p = 0.004), ODO2 (2.04 fold increase, *p* = 0.006), IDH3A (twofold increase, *p* = 0.011), NU5M (1.53 fold increase, *p* = 0.009), GRP75 (4.18 fold increase, *p* = 0.03), SCOT1 (3.6 fold increase, *p* = 0.01), ADT1 (0.38 fold increase, *p* = 0.04), ATPK (2.62 fold increase, *p* = 0.035), ATP5E (4.36 fold increase, *p* = 0.0067), EHCM (2.15 fold increase, *p* = 0.007). Other proteins changing in the parahippocampus, not related to the mitochondria, included UCLH1, TDP43, cold inducible RNA binding protein, ATP13A2, AATC. Changes in a mildly affected region, at the boundary of pathology vs no pathology, reflect alterations in pathways that occur early in the disease process, here indicated to be mitochondrial metabolism, bioenergetics, ATP synthesis, and the mitochondrial stress response.

**Table 3:**
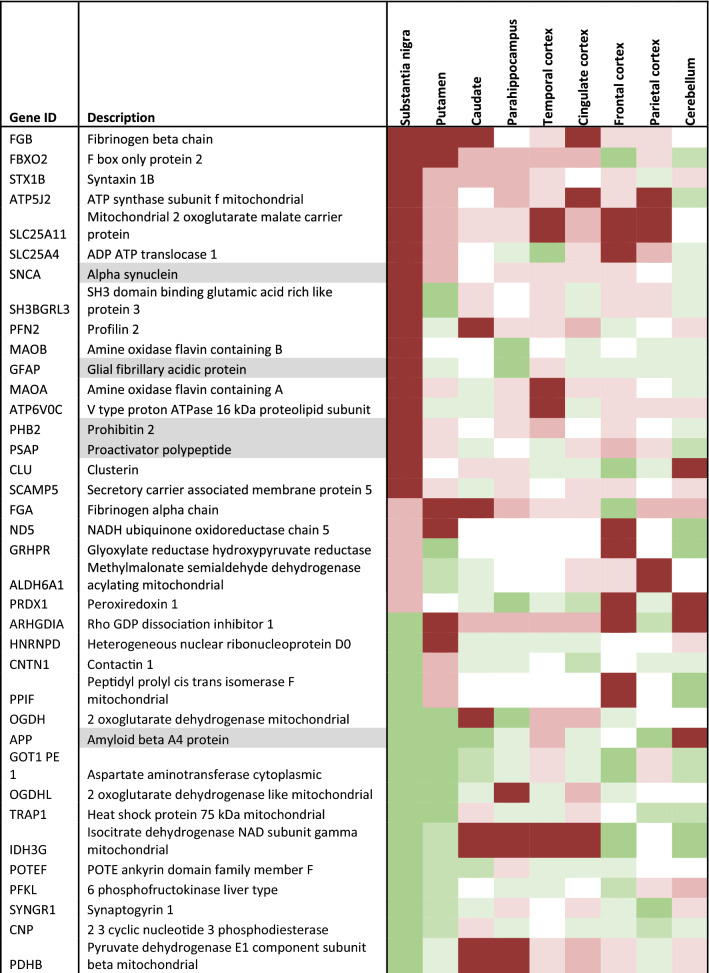

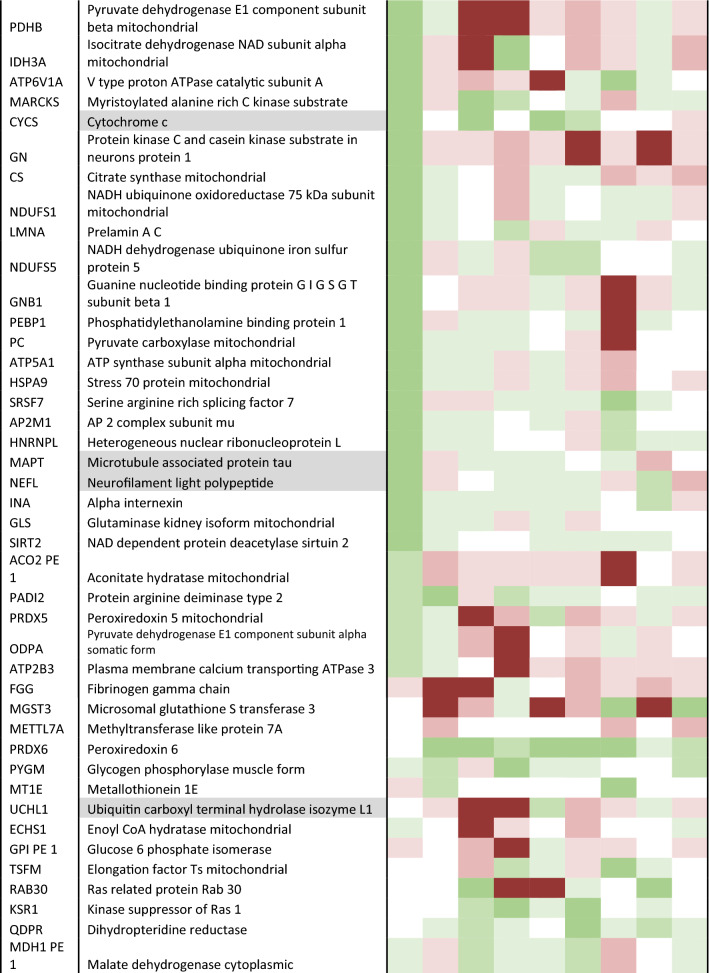

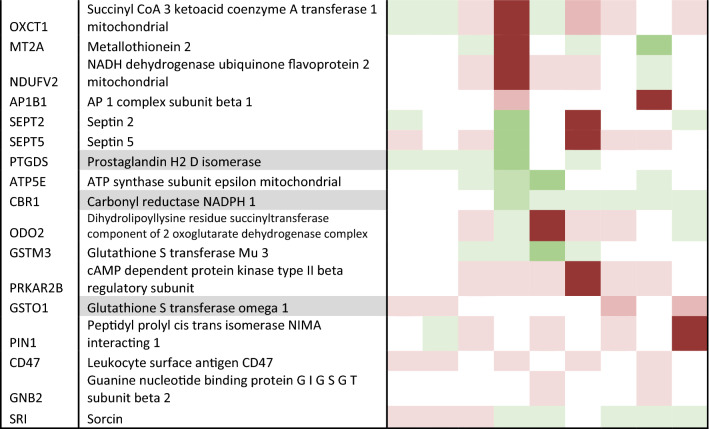
Candidate list of proteins to validate

Description is shaded if from the insoluble fraction. Heatmap shows level of up or down regulation for each protein in each brain region in early PD (Braak stage 3/4) compared to controls. Red indicates upregulation in PD and green indicates downregulation with intensity of colour indicating level of expression change

Certain proteins demonstrated changes across several brain regions with varying pathology. As shown in Fig. 4, metallothionein-2 showed an increase in regions with moderate pathology (putamen, *p* = 0.03), mild pathology (cingulate cortex, *p* = 0.022; parahippocampus, *p* = 011) and no pathology (frontal cortex, *p* = 0.008). Mitochondrial 2-oxoglutarate malate carrier exhibited a fivefold increase in a region with moderate pathology (putamen, *p* = 0.03), a 4.7 fold increase in a region with mild pathology (parahippocampus, *p* = 0.01) and a 1.8 fold increase in a second region with mild pathology (cingulate cortex, *p *= 0.01), Fig. 4p. Microsomal glutathione S transferase demonstrated changes in a moderate pathology region, putamen (2.93, *p* = 0.04), as well in the mildly affected regions, cingulate cortex (0.99, *p* = 0.006), and temporal cortex (1.54, *p* = 0.0025). Such proteins suggest pathways affecting multiple regions susceptible to PD, independent of disease stage, may include mitochondrial metabolism, redox balance, and metal ion homeostasis.

Proteins that showed statistically significant changes in the frontal and parietal cortex (no pathology at this early-mid stage, but predicted to be vulnerable in PD), may highlight the very earliest changes in cells seen before the emergence of a synucleinopathy. These include Sirtuin-2, metallothionein 2, and glycogen phosphorylase.

Proteins that were highlighted to change (HADHB, ACAT1, VDAC2, IDH3A, GPR75, SIRT2, G6PD, ATP13A2, NDUFA2, SLC25A11, ACO2, DLST, OXCT1, SLC25A4, NDUFS5, ATP5MF, ATP5F1E, ECHS1, MT2A, TARDBP, GOT1, MGST1, PYGL) were mined in the available cell specific datasets, and those detected are shown in Additional file 8: Table S5. Protein enrichment for the selected targets (23) were analysed using the GeneOverlap Bioconductor package in cell types and a significant association was found with Sharma et al (2015) oligodendrocyte dataset (FDR = 0.03) with overlap occurring for 3 target proteins: SIRT2, ACAT1 and PYGL. Other proteins overlapped with additional other cell types e.g MT2A overlapped with astrocyte cell type data (Additional file 8: Table S5) but these were non-significant. These results indicate that further investigation to determine how these key proteins act within different cell types is needed.

### Detailed characterisation of the mitochondrial proteome in 4 stages of disease

**Fig. 5:**
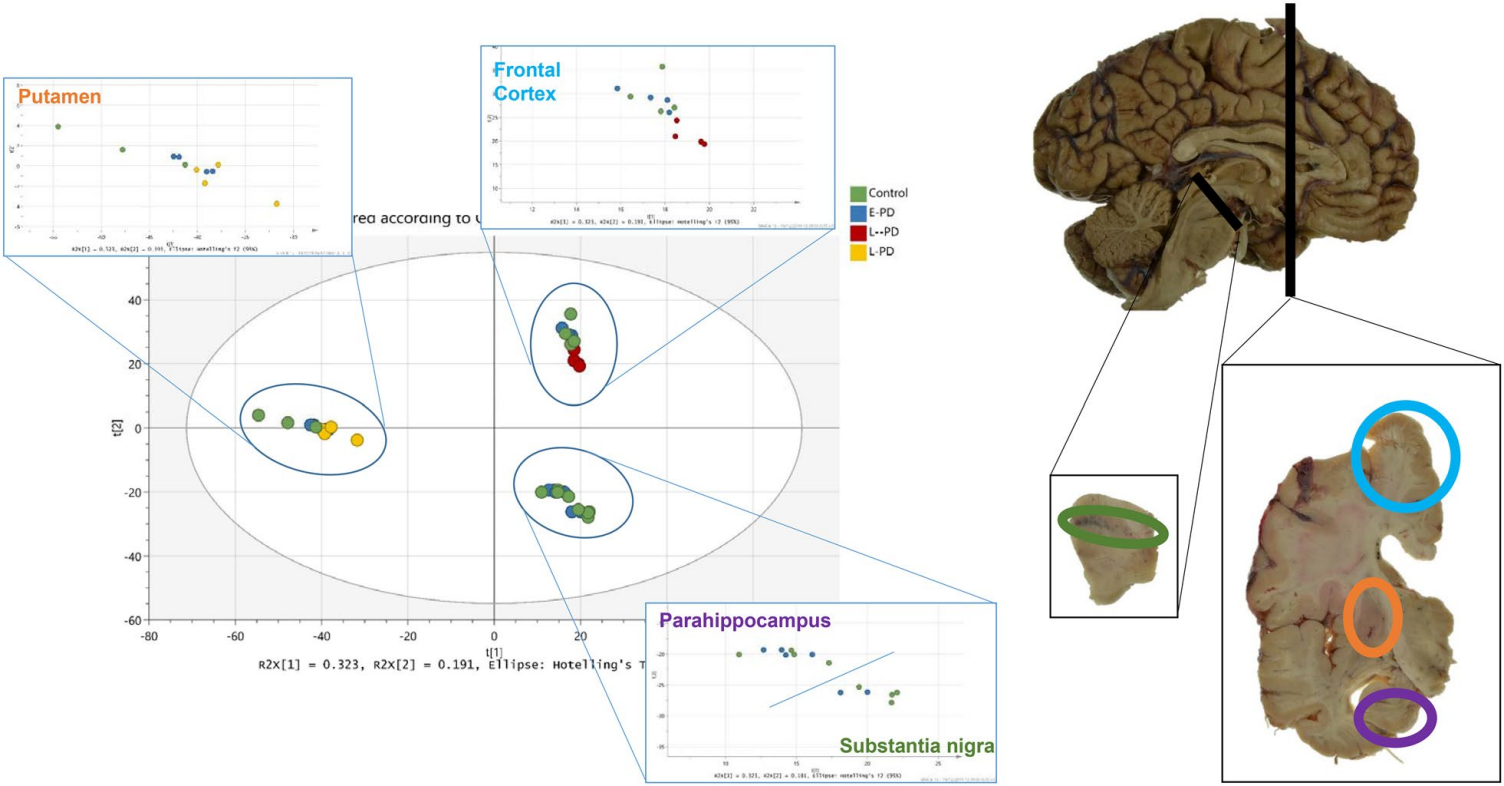
Principle component plot of Synapt G2-Si data. Variation is accounted primarily by brain region. Sub region variation can be observed between late PD and control and early PD but no overall proteomic variation is observed between control and early PD indicating the changes are subtle. Each brain region is mapped on hemi-dissected hemisphere and coronal slice, in its respective annotated colour

We sought to understand in greater depth, the nature of the mitochondrial dysfunction in early-mid post-mortem brain. Therefore, we repeated the HPLC–ESI–MS/MS analysis on the higher resolution Synapt-G2 Si machine, using a sub-selection of the original samples, performed individually across 4 selected regions: substantia nigra, putamen, parahippocampus and frontal cortex (representing severe, moderate, mild and unaffected regions). We detected a combined (supernatant and pellet) total of 2343 proteins. A principal components analysis (Fig. 5) shows that the variation between samples is primarily accounted for by brain region. Furthermore, the differences in the proteome between control and early PD are modest, suggesting that early disease varies subtly from control brain.

In the first analysis (pooled samples, 8 PD brain regions), we illustrated that 202 proteins with altered expression (> 1.5 fold) in early PD vs control were associated with mitochondria (human MitoCarta 2.0 [42]). In the second analysis (individual samples, 4 brain regions), 140 mitochondrial proteins exhibited a change in expression (> 1.5 fold) in early PD vs controls (79 of these overlapped with the proteins identified in the first analysis). The second data set (2–4 individuals, 4 brain regions) was analysed using the IPA software. The three most significantly changing pathways across the 4 regions were ‘mitochondrial dysfunction’, ‘oxidative phosphorylation’, and ‘sirtuin signalling’, thereby replicating our original finding (Fig. 3b). The altered expression of mitochondrial proteins in each region are shown in Table 4 and as a heatmap in Additional file 1: Figure S1c.

**Table 4:** Mitochondrial proteins that show altered expression

ID	Name	SN	Put	Para	Fron
ACO1	Aconitase 1	1.017	1.105	1.725	1.036
ACO2	Aconitase 2	1.218	− 1.131	1.186	1.252
AIFM1	Apoptosis inducing factor mitochondria associated 1	1.219	1.273	− 1.072	1.246
ATP5F1A	ATP synthase F1 subunit alpha	1.748	− 1.437	1.243	1.217
ATP5F1B	ATP synthase F1 subunit beta	2.617	1.057	1.091	1.37
ATP5F1C	ATP synthase F1 subunit gamma	3.771	− 1.132	− 1.386	1.383
ATP5F1D	ATP synthase F1 subunit delta	− 1.096	1.168	− 1.281	1.208
ATP5ME	ATP synthase membrane subunit e	1.53	− 1.019	1.18	− 1.395
ATP5MF	ATP synthase membrane subunit f	− 1.163	− 1.207	1.19	1.089
ATP5MG	ATP synthase membrane subunit g	1.452	1.015	1.068	1.145
ATP5PB	ATP synthase peripheral stalk-membrane subunit b	2.056	− 1.114	1.256	1.041
ATP5PD	ATP synthase peripheral stalk subunit d	− 1.051	− 1.153	1.328	1.137
ATP5PF	ATP synthase peripheral stalk subunit F6	− 1.599	1.073	1.265	1.11
ATP5PO	ATP synthase peripheral stalk subunit OSCP	2.228	− 1.136	1.251	1.247
ATPAF2	ATP synthase mitochondrial F1 complex assembly factor 2	− 2.474	1.373	1.024	1.221
CAT	Catalase	1.599	− 1.591	1.273	− 1.07
COX4I1	Cytochrome c oxidase subunit 4I1	1.209	− 1.057	1.389	1.403
COX5A	Cytochrome c oxidase subunit 5A	− 1.4	− 1.124	1.32	1.476
COX5B	Cytochrome c oxidase subunit 5B	1.15	− 1.009	1.169	1.255
COX6A1	Cytochrome c oxidase subunit 6A1	1.39	− 1.198	− 1.158	1.306
COX6B1	Cytochrome c oxidase subunit 6B1	− 1.107	1.311	− 1.683	2.323
COX7A2	Cytochrome c oxidase subunit 7A2	1.037	− 1.333	− 1.694	1.367
COX7C	Cytochrome c oxidase subunit 7C	− 1.601	− 1.15	− 1.115	1.193
CYB5R3	Cytochrome b5 reductase 3	1.053	− 1.134	− 1.002	1.042
CYC1	Cytochrome c1	1.512	− 1.055	1.381	1.375
CYCS	Cytochrome c, somatic	1.358	1.085	1.091	1.62
FIS1	Fission, mitochondrial 1	− 1.254	− 1.043	1.106	− 1.093
GPD2	Glycerol-3-phosphate dehydrogenase 2	1.69	1.06	1.166	1.016
GPX4	Glutathione peroxidase 4	1.505	− 1.039	1.035	− 1.114
GSR	Glutathione-disulfide reductase	− 1.592	− 1.247	1.276	1.781
HSD17B10	Hydroxysteroid 17-beta dehydrogenase 10	− 1.79	− 1.16	1.149	− 1.032
HTRA2	HtrA serine peptidase 2	1.414	1.032	1.047	1.123
MAOA	Monoamine oxidase A	1.055	− 1.118	1.102	1.213
MAOB	Monoamine oxidase B	1.543	− 1.111	− 1.253	− 1.089
MAP2K4	Mitogen-activated protein kinase inase 4	1.469	1.163	1.155	− 1.012
MAPK9	Mitogen-activated protein kinase 9	− 9.433	NA	1.238	− 1.14
MT-ATP6	ATP synthase F0 subunit 6	2.2	− 2.204	− 16.992	1.588
MT-CO1	Cytochrome c oxidase subunit I	1.233	− 1.936	NA	NA
MT-CO2	Cytochrome c oxidase subunit II	2.063	− 1.129	1.227	1.577
MT-ND4	NADH dehydrogenase, subunit 4 (complex I)	5.659	− 1.082	1.048	1.167
NDUFA10	NADH:ubiquinone oxidoreductase subunit A10	1.391	− 1.067	1.046	1.499
NDUFA11	NADH:ubiquinone oxidoreductase subunit A11	1.174	− 1.357	1.579	1.293
NDUFA12	NADH:ubiquinone oxidoreductase subunit A12	1.45	− 1.196	1.234	1.194
NDUFA13	NADH:ubiquinone oxidoreductase subunit A13	− 1.378	1.085	1.526	2.629
NDUFA2	NADH:ubiquinone oxidoreductase subunit A2	1.301	− 1.154	1.166	− 1.121
NDUFA3	NADH:ubiquinone oxidoreductase subunit A3	4.303	1.12	− 2.074	− 1.255
NDUFA4	NDUFA4 mitochondrial complex associated	1.808	− 1.156	1.54	1.187
NDUFA5	NADH:ubiquinone oxidoreductase subunit A5	1.133	− 1.19	1.183	1.425
NDUFA6	NADH:ubiquinone oxidoreductase subunit A6	1.479	− 1.104	1.212	2.249
NDUFA7	NADH:ubiquinone oxidoreductase subunit A7	1.192	1.044	1.254	− 1.507
NDUFA8	NADH:ubiquinone oxidoreductase subunit A8	1.047	− 1.109	− 1.994	1.062
NDUFA9	NADH:ubiquinone oxidoreductase subunit A9	1.442	− 1.069	1.245	1.297
NDUFB10	NADH:ubiquinone oxidoreductase subunit B10	1.506	1.038	1.131	1.198
NDUFB11	NADH:ubiquinone oxidoreductase subunit B11	− 1.076	− 1.018	1.005	1.448
NDUFB3	NADH:ubiquinone oxidoreductase subunit B3	1.592	1.025	1.074	1.228
NDUFB4	NADH:ubiquinone oxidoreductase subunit B4	− 1.122	− 1.039	1.156	1.261
NDUFB6	NADH:ubiquinone oxidoreductase subunit B6	3.766	1.078	− 5.285	− 1.123
NDUFB7	NADH:ubiquinone oxidoreductase subunit B7	1.091	2.52	NA	NA
NDUFB9	NADH:ubiquinone oxidoreductase subunit B9	− 1.002	1.031	1.203	1.278
NDUFS1	NADH:ubiquinone oxidoreductase core subunit S1	1.415	− 1.151	1	1.147
NDUFS2	NADH:ubiquinone oxidoreductase core subunit S2	5.867	− 1.019	-1.016	1.061
NDUFS3	NADH:ubiquinone oxidoreductase core subunit S3	1.224	1.038	1.163	1.445
NDUFS4	NADH:ubiquinone oxidoreductase subunit S4	1.384	1.062	1.099	− 2.262
NDUFS5	NADH:ubiquinone oxidoreductase subunit S5	1.534	− 1.249	− 1.324	1.281
NDUFS6	NADH:ubiquinone oxidoreductase subunit S6	− 1.329	− 1.261	− 1.138	1.254
NDUFS7	NADH:ubiquinone oxidoreductase core subunit S7	1.621	− 1.274	1.071	1.48
NDUFS8	NADH:ubiquinone oxidoreductase core subunit S8	1.521	− 1.017	1.046	1.271
NDUFV1	NADH:ubiquinone oxidoreductase core subunit V1	1.301	− 1.153	1.011	1.521
NDUFV2	NADH:ubiquinone oxidoreductase core subunit V2	− 1.217	− 1.056	1.593	1.554
OGDH	Oxoglutarate dehydrogenase	− 1.284	1.071	− 1.097	1.123
PARK7	Parkinsonism associated deglycase	1.405	− 1.064	− 1.18	− 1.059
PDHA1	Pyruvate dehydrogenase E1 alpha 1 subunit	− 1.167	− 1.565	1.106	1.253
PRDX3	Peroxiredoxin 3	1.393	1.08	1.154	1.267
PRDX5	Peroxiredoxin 5	− 1.19	− 1.184	− 1.11	1.079
RHOT2	Ras homolog family member T2	− 1.367	NA	NA	NA
SDHA	Succinate dehydrogenase complex flavoprotein subunit A	1.492	1.009	1.202	− 1.412
SDHB	Succinate dehydrogenase complex iron sulfur subunit B	1.519	− 1.121	− 1.056	1.116
SNCA	Synuclein alpha	2.154	− 1.311	1.229	1.091
SOD2	Superoxide dismutase 2	1.485	− 1.35	− 1.073	1.215
TXN2	Thioredoxin 2	− 1.908	NA	NA	NA
UQCRB	Ubiquinol-cytochrome c reductase binding protein	− 1.15	− 1.03	1.17	− 1.267
UQCRC1	Ubiquinol-cytochrome c reductase core protein 1	2.346	− 1.294	− 1.469	1.473
UQCRC2	Ubiquinol-cytochrome c reductase core protein 2	1.29	− 1.138	− 1.297	1.141
UQCRFS1	Ubiquinol-cytochrome c reductase, Rieske iron-sulfur polypeptide 1	− 1.21	− 1.002	− 1.01	1.191
UQCRH	Ubiquinol-cytochrome c reductase hinge protein	− 1.691	NA	− 3.694	NA
UQCRQ	Ubiquinol-cytochrome c reductase complex III subunit VII	2.212	1.027	1.057	1.069
VDAC1	Voltage dependent anion channel 1	2.691	− 1.067	1.352	1.057
VDAC2	Voltage dependent anion channel 2	1.57	− 1.004	1.053	1.209
VDAC3	Voltage dependent anion channel 3	1.723	− 1.207	1.055	1.208

All proteins involved in mitochondrial dysfunction pathway (as determined by IPA) and the fold change expression for each in early PD compared to controls per region

**Fig. 6:**
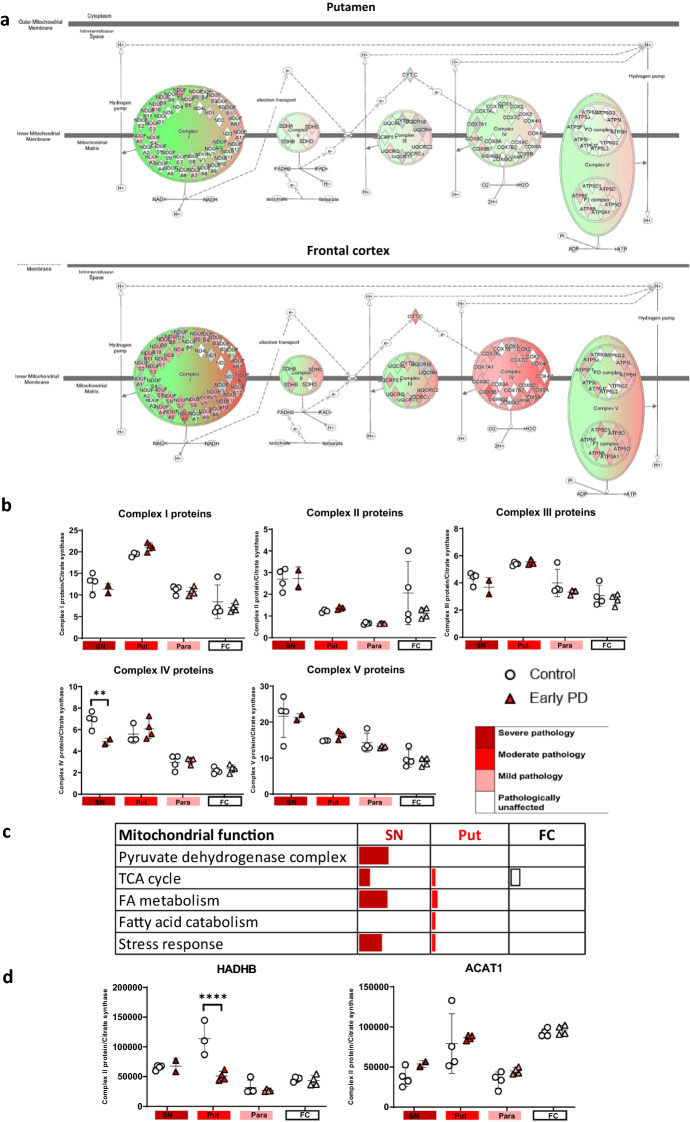

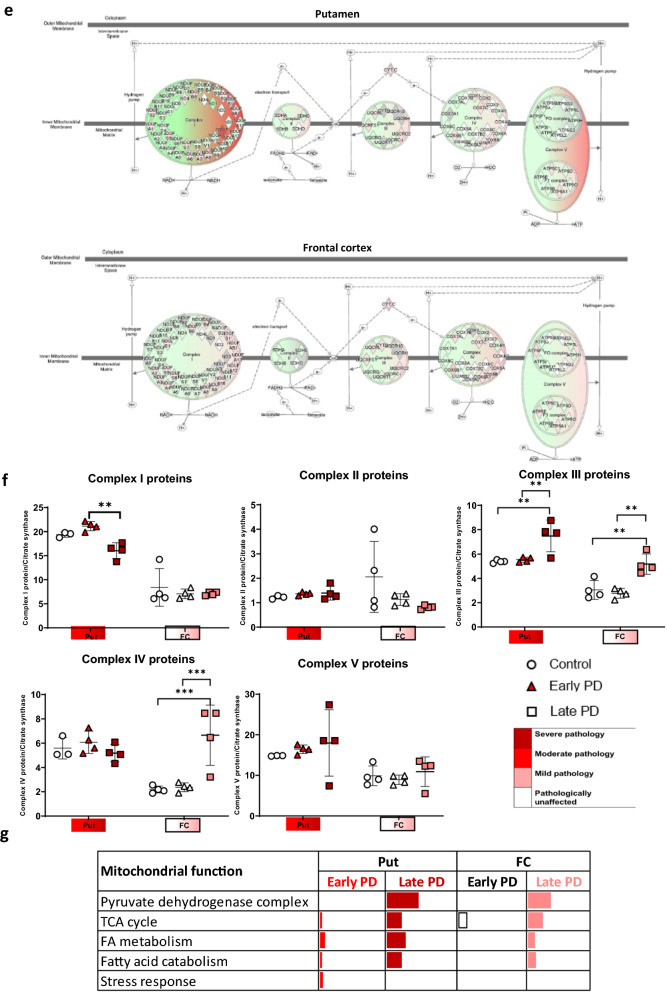
Mitochondrial analysis of label free proteomic data. Oxidative phosphorylation pathway diagram with per region overlay of proteomic expression for the putamen and frontal cortex in early PD compared to controls, **a** and late PD compared to controls, **e**. Red represents upregulation and green represents downregulation. Intensity of colour indicates level of altered expression. **b**, **f** Complex proteins totalled and ratioed to citrate synthase across brain regions and disease stages. Two-way ANOVA with Sidek’s multiple comparison post-hoc testing was performed for each complex/citrate synthase and statistically significant results are demonstrated on graphs with * representing *p* < 0.03, ** representing *p* < 0.002, *** representing *p* < 0.0002 and **** representing *p* < 0.0001. Bars represent SEM. **c**, **g** Table indicating the % of significantly altered proteins per pathway for the sub-functions of the mitochondria as determined through Mitominer and Panther. Regions are colour-coded for severity of pathology present at that stage of disease as shown in key. **d** HADHB and ACAT1 expression across multiple brain regions, Graphs and statistics were completed using GraphPad Prism v8

The mitochondrial proteome was further analysed to assess the specific mitochondrial processes affected. Mitochondrial proteins were sub-grouped into key mitochondrial functions and the percentage of significantly affected proteins in each function is illustrated in Fig. 6c and g. Regions with mild and severe pathology (late frontal cortex, and putamen of early/late PD), demonstrated changes in the ‘pyruvate dehydrogenase complex’, ‘TCA cycle’, ‘Fatty acid catabolism’, and ‘stress response’. Interestingly, regions with no pathology (early frontal) still exhibited alterations in the TCA cycle. Fatty acid oxidation is not the primary source of energy in the brain, however, neuronal cells utilise fatty acids as fuel when in a diseased state [43]. These changes may indicate altered metabolic flux of energy substrates, such as acetyl-coA. Fatty acid oxidation is associated with increased expression of ACAT1, which is increased in the early frontal cortex as well as the late frontal cortex. This indicates that there may be a switch in the metabolic use towards fatty acid oxidation. Notably, we also observe decreased expression of mitochondrial trifunctional enzyme subunit beta (HADHB), in early and late PD putamen, compared to controls (Fig. 6d).

Mitochondrial complex protein abundance values were extracted, totalled, and ratioed to protein abundance of citrate synthase to compare between conditions (Fig. 6b and f). Notably, an upregulation of complex I, II and V occurs in early putamen. Changes in complex II and/or IV appear to only occur in late putamen and frontal cortex. To determine if changes could be due to variation in mitochondrial number, the levels of citrate synthase were examined. Citrate synthase levels did not change between disease groups (*p* = 0.1647) but did alter between regions (*p* < 0.0001, Additional file 3: Figure S3).

Overall, this data demonstrates that multiple mitochondrial pathways may be simultaneously affected in PD, and these include the TCA cycle and fatty acid oxidation, as well as oxidative phosphorylation and ATP synthesis.

### Mitochondrial pathology worsens with disease progression

**Fig. 7:**
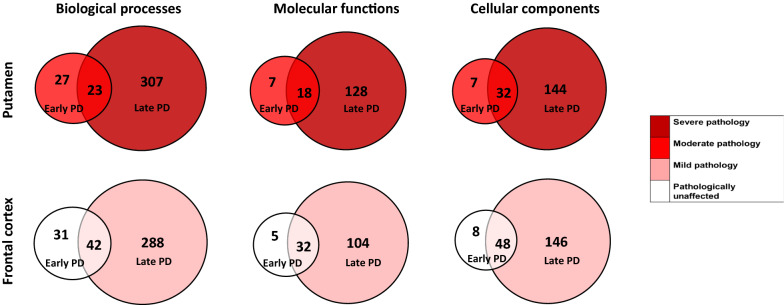
Early stage PD overlap against Late stage PD. Pie charts show the number of GO terms (Biological processes, molecular functions or cellular components) that are overlapping between early PD (Braak stage 3/4) and late stage PD (Braak stage 6) in either the putamen, a region moderately affected at Braak stage 3/4 but severely affected at Braak stage 6, and the frontal cortex, a region not affected at Braak stage 3/4 but mildly affected at Braak stage 6. Colour coded for severity of pathology present at that stage of disease as shown in key

In addition to assessing Braak stage 3/4 brains, we assessed late Braak stage 6 PD brain (compared to controls) for two selected regions—the frontal cortex and the putamen, shown in the PCA plot (Fig. 5). This enabled us to study the same region across two disease stages: that is, the frontal cortex unaffected (Braak stage 3, termed early frontal) and mildly affected (Braak stage 6, termed late frontal). We studied the overlap between the early brain changes (early vs control) and the late brain changes (late vs control) (Fig. 7). Together, this analysis demonstrated that the progression to late stage brain, within a region, is associated with common pathways to early PD brain, and the acquisition of more, and new pathways in late PD.

Exclusive to early frontal cortex where no pathology is observed, are ‘mitochondrion organisation’, ‘protein localisation to mitochondrion’ and ‘reactive oxygen species metabolism’ (Additional file 10: Table S7). Overlapping between early frontal cortex and late frontal cortex includes ‘response to reactive oxygen species’, ‘mitochondrial electron transport’, ‘mitochondrial complex 1’, and ‘mitochondrial apoptosis’. Exclusive to late frontal cortex includes ‘mitochondrial fission’, ‘release of cytochrome c from mitochondria’, ‘acetyl-coA and pyruvate metabolism’, ‘TCA cycle’, and ‘mitochondrial electron transport’.

The overlapping BPs in early putamen (Braak stage 3) vs late putamen (Braak stage 6) included ‘response to reactive oxygen species’, ‘fatty acid beta-oxidation’ and ‘insulin receptor signalling pathway’ (Additional file 11: Table S8). Exclusive to the late stage putamen includes ‘mitochondrial genome maintenance’, ‘mitochondrial fission’, ‘acetyl-CoA metabolism’, ‘pyruvate metabolism’, ‘TCA cycle’, and ‘mitochondrial electron transport’. This suggests the existence of mitochondrial pathway disruption early in putamen pathology, with progression to prominent and persistent mitochondrial dysfunction in late putamen pathology.

Together, this data suggests that mitochondrial pathways are disrupted in unaffected regions in early PD, and that these processes progress to affect further mitochondrial and metabolic pathways over the course of the disease.

### Validation of mitochondrial activity in PD brain

**Fig. 8:**
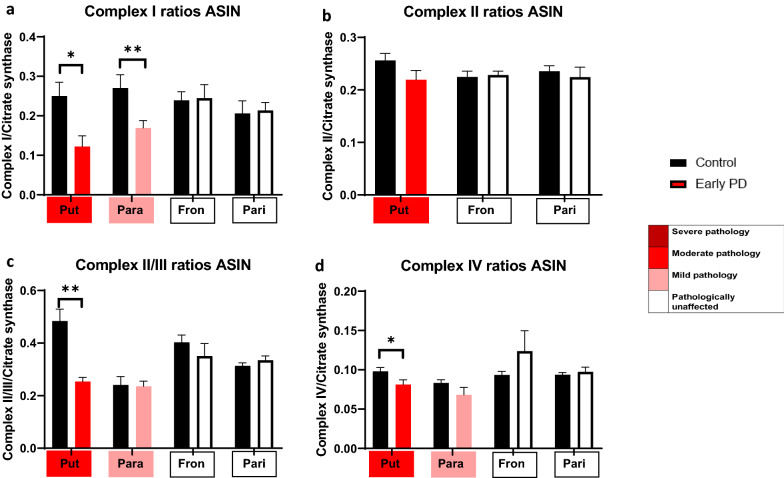
Mitochondrial assays show altered mitochondrial function in early affected regions. Functional mitochondrial assays across the putamen, parahippocampus, frontal cortex and parietal cortex for **a** Complex I, **b** Complex II, **c** Complex II/III and **d** Complex IV. All are normalised to citrate synthase levels and the ArcSin (ASIN) of data used for statistical analysis. Regions are colour-coded for severity of pathology present at that stage of disease as shown in key. Mann–Whitney U-tests were performed with * representing *p* < 0.03, ** representing *p* < 0.002, *** representing *p* < 0.0002 and **** representing *p* < 0.0001. Error bars represent SEM. Graphs and statistics were completed using GraphPad Prism v8

Due to the high prevalence of pathways involving mitochondrial metabolism and ATP synthesis, we reasoned that mitochondrial dysfunction may be detectable in early PD brain. Respiratory chain activity can be studied in post-mortem brain, and has been previously implicated in PD pathology, primarily relating to complex I dysfunction [44, 45]. We took four brain regions reflecting moderate pathology, mild pathology, and no pathology (putamen, parahippocampal cortex, frontal cortex, and parietal cortex), and investigated their respiratory chain activity, normalising to citrate synthase (Fig. 8). In the moderately affected region in this study (putamen), we found a significant decrease in complex I activity (*p* = 0.026), complex II plus III activity (*p* = 0.0043), and in complex IV activity (*p* = 0.0281). Interestingly, this reveals complete impairment of the respiratory chain. Notably, this decrease in complex activity was associated with an increase in complex protein levels (Fig. 6b and f), perhaps indicative of a compensatory mechanism or that the increased complex protein is damaged and non-functional. In the mildly affected cortical region (parahippocampal cortex), we found a significant reduction in complex I function only (*p* = 0.0079), with preservation of the other complexes. However, in unaffected regions (frontal and parietal cortex) we found no impairment of respiratory chain activity, whilst isolated complex II activity was unaffected in all cases. This data demonstrates that in early PD brain, the appearance of respiratory chain dysfunction and metabolism occurs in association with mild aggregation pathology and is associated with a progressive and severe respiratory chain dysfunction in mildly and moderately affected regions.

## Discussion

This study identifies critical proteins and pathways altered in early sporadic PD. Importantly, we focus on Braak stage 3–4 brain, in which LB pathology and neuronal loss are found on a gradient from severe-moderate-mild-unaffected [5]. Sampling across this gradient allows the capture of the early molecular changes as well as the late molecular changes resulting from both PD pathology and the bystander effects of neuronal loss. Here, we performed an unbiased capture of data across the PD brain, harnessing the spatio-temporal gradient of pathology, to investigate 2 key disease signatures: (i) common proteins and pathways across disease regions independent of pathological stage, thus reflecting shared causal mechanisms and/or shared molecular signatures of cell vulnerability, (ii) proteins and pathways with altered expression in regions with mild or unaffected pathology, likely reflecting the early drivers of the disease.

Proteomics provides large scale data of protein expression in complex biological samples, enabling the inference of biologically meaningful functional information. However, tissue heterogeneity and technical variability has led to a lack of consistency and reproducibility [46]. Here, punch dissection of carefully delineated anatomical regions enabled precision of sampling to the level of cortical areas and brain nuclei, whilst still capturing multiple cell types in a region [22] and retaining sufficient throughput. Our proteomic data confirmed that the expression of insoluble alpha-synuclein in these brains existed in the same biochemical gradient as that characterised immunohistochemically by LB pathology. We demonstrate that the number of altered biological processes or molecular function terms matches the severity of pathology. As expected, the highest number of altered pathways occurs in severely affected regions, with the smallest number found in mild or unaffected regions. The spatio-temporal gradient of pathology in early PD brains is thus matched by a biochemical gradient of a changing proteome. This observation further supports the approach of studying the small number of altered pathways from pathologically unaffected or mildly affected regions to understand the driving events in the disease process.

### Identifying common pathways across early PD brain

Ingenuity pathway analysis revealed mitochondrial dysfunction as the ‘top pathway’ in early PD, with altered expression across all regions. The comparison of common pathways across the early PD brain, filtering for proteins with a fold change > 1.5 and common to > 5 brain regions, resulted in the clear emergence of mitochondrial proteins (33% of total) and mitochondrial pathways such as metabolism, oxidative phosphorylation, and oxidative stress. Next, we compared two specific brain regions at different stages of disease: (i) the frontal cortex of Braak stage 3 (unaffected) versus Braak stage 6 (mildly affected), in which the convergent processes in the same region with different stages of pathology included mitochondrial dysfunction. (ii) the putamen from Braak stage 3 (moderate pathology) versus Braak stage 6 (severe pathology) also demonstrated mitochondrial metabolism and oxidative stress as convergent pathways. These approaches, that is, within early PD brain across pathology stages, as well as across PD brain of different stages, highlight the importance of mitochondrial function early in the disease, prior to pathology, and in late disease.

Specific mitochondrial located proteins that demonstrated clearly altered expression in a single or multiple affected regions of the early stage brain in both proteomic studies and the validation study predict critical processes that are affected in early PD:

(i) **Oxidative phosphorylation and ATP synthesis**, due to specific changes in components of the respiratory chain, notably NADH-ubiquinone oxidoreductase chain 5 (Core subunit of the mitochondrial membrane respiratory chain NADH dehydrogenase (Complex I)), and NADH dehydrogenase (ubiquinone) 1 alpha subcomplex subunit 2 (Accessory subunit of the mitochondrial membrane respiratory chain NADH dehydrogenase (Complex I)), ATP synthase F(0) complex subunit B1, and ATP synthase subunit epsilon (both key components of mitochondrial membrane ATP synthase (F1F0 ATP synthase or Complex V) that produces ATP from ADP).
(ii) **Mitochondrial metabolism,** due to specific changes in Isocitrate dehydrogenase (NAD) subunit alpha (Catalytic subunit of the enzyme which catalyses the decarboxylation of isocitrate (ICT) into alphaketoglutarate); Succinyl-CoA:3-ketoacid coenzyme A transferase 1 (Key enzyme for ketone body catabolism which transfers the CoA moiety from succinate to acetoacetate), Dihydrolipoyllysine-residue succinyltransferase component of 2-oxoglutarate dehydrogenase complex (The 2-oxoglutarate dehydrogenase complex catalyses the overall conversion of 2-oxoglutarate to succinyl-CoA and CO2), Aconitate hydratase (Catalyses the isomerisation of citrate to isocitrate via cis-aconitate), Mitochondrial 2-oxoglutarate malate carrier (Catalyses the transport of 2-oxoglutarate across the inner mitochondrial membrane in an electroneutral exchange for malate or other dicarboxylic acids). Other affected mitochondrial proteins include the Voltage dependent anion-selective channel protein 2, which mediates transport across the mitochondrial outer membrane, and Stress-70 protein, a mitochondrial chaperone protein, and ADP/ATP translocase which mediates the transport of ATP out of the mitochondria. Interestingly, Microsomal glutathione S-transferase 3, changed its expression across 3 PD regions, and is the enzyme that catalyses oxidation of hydroxy-fatty acids, and participates in lipid metabolism.
(iii) **Glutathione metabolism and redox regulation**, glucose-6-phosphate dehydrogenase (G6PD) is reduced in early frontal cortex. G6PD is involved in the pentose-phosphate pathway and reduces glutathione [47]. This is in line with previous studies that have commented on the pentose-phosphate pathway involvement in PD and identified reduction in G6PD in the putamen of PD post-mortem brain [48]. Reduced Glutathione combats and protects against oxidative stress [49, 50]. The enzyme that catalyses this reaction, glutathione reductase is also reduced in the substantia nigra and putamen. Glutathione-S-transferase omega-1 is also reduced in early putamen and early frontal cortex. This enzyme is responsible along with glutathione for protecting the mitochondrial membranes from oxidative damage [51]. Alteration of this pathway early in disease would explain why an increase in oxidative stress is observed. As a consequence of increased oxidative stress, protein mixed disulfides can form and this can cause a number of adverse effects for other proteins and the way they function [52]. Additionally oxidative stress can reduce transcription of TCA cycle enzymes [53]. Hence the disruption of these pathways in early PD may lead to increased mitochondrial dysfunction and progression of disease.

Notably, respiratory chain enzyme activity can be assessed in frozen post-mortem tissue, with this approach leading to the discovery that impaired complex I function is associated with PD pathology [44, 45]. Here, we discovered that regions of moderate pathology, including the putamen, are associated with severe and comprehensive functional abnormality, affecting all complexes of the respiratory chain. In areas of mild pathology, such as the parahippocampus, in which significant changes of mitochondrial proteins, including complex I were validated, only complex I is significantly impaired. Conversely, in pathologically unaffected regions, which were associated with minimal changes in mitochondrial located proteins, no functional deficit was observed. Therefore, we concluded that in early stage PD brain, the mitochondrial respiratory chain function follows a gradient of activity from no impairment, to severely impaired, along the gradient of α-synuclein pathology. Interestingly, the primary induction of complex I dysfunction in mice results in reduced dopamine synthesis and motor deficits [54], further highlighting the likely importance of mitochondrial bioenergetic impairment early in the disease. It should be noted that no loss of isolated complex II activity was demonstrated in this study. In areas with reduced complex activity, we observed an increase in expression levels of complexes. It is therefore possible that the presence of oxidative stress leads to post translational modifications that damage the protein complexes, altering their function. Further work is required to ascertain why this exclusively nuclear encoded component of the respiratory chain is preserved, i.e. the susceptibility of nuclear vs mitochondrial DNA and their gene products, to oxidative stress [55].

Nigral alterations in mitochondrial respiration and oxidative damage have been previously identified in post-mortem brain [56, 57]. Analysis by Werner et al [58]. of the substantia nigra, of 5 PD cases, detected 321 differentially expressed proteins, including those involved in iron metabolism and glutathione related redox metabolism. Another study of the substantia nigra found 119 differentially expressed proteins in PD patients, including proteins involved in mitochondrial dysfunction, oxidative stress, protein degradation, and neuroinflammation [15]. Notably, oxidative damage is not limited to the substantia nigra in post-mortem PD brain, but is evident in the frontal cortex, with proteins such as alpha-synuclein, UCLH1, SOD1, SOD2, and DJ-1 undergoing oxidative modifications [59, 60]. Furthermore, early stage PD has been associated with oxidatively modified proteins implicated in glycolysis and energy metabolism [61]. Therefore, the current literature suggests that mitochondrial dysfunction and oxidative stress are critical features of PD pathology.

Our data confirms that alterations in mitochondrial pathways and function are key features of the PD brain. Importantly, we find that significant changes in mitochondrial protein expression occur early in the disease process in pathologically unaffected regions (frontal and parietal cortex), indicating the likely role of mitochondrial dysfunction in PD pathogenesis. We next examined further changes in unaffected regions that may occur prior to the development of alpha-synuclein pathology. Our study validated metallothionein-2 and sirtuin-2 as 2 proteins that change significantly in regions unaffected pathologically, but predicted to be affected in PD. Metallothionein-2 has a high content of cysteine residues that bind various heavy metals, altering the bioavailability of metals associated with PD, such as iron, copper, and zinc [62, 63]. Interestingly, both cell and animal models have demonstrated that metallothionein-2 protects against the nitrative and peroxynitrite stress induced by alpha-synuclein [64, 65], whilst its treatment prevents paraquat-induced loss of dopaminergic neurons [66]. We show an overlap of MT2A in astrocyte proteomic cell type data. It has been previously shown to be expressed in astrocytes and further to be transferred to neurons during the process of axon regeneration [67, 68]. Here, metallothionein-2 exhibited increased expression along the gradient of pathology in the early stage PD brain and remained elevated in unaffected frontal cortex. This highlights a protein involved in heavy metals and oxidative stress as playing a key role in the early stages of PD and indicates the potential for astrocytic involvement in early disease processes.

NAD-dependent protein deacetylase sirtuin-2 is considered a master regulator of ageing processes, modulating multiple and diverse biological processes through the deacetylation of internal lysines on many proteins. Sirtuin-2 is implicated in a number of pathways critical in PD pathogenesis, including mitochondrial metabolism, mitophagy, autophagy, and oxidative stress [69–74]. Furthermore, sirtuin-2 has been shown to deacetylate alpha-synuclein, driving its aggregation and toxicity [75]. Thus, the inhibition or deletion of sirtuin-2 is shown to be neuroprotective in PD models [76–78]. However, reduced sirtuin-2 has also been shown to have deleterious effects [73, 79, 80], demonstrating that whilst its loss may reduce alpha-synuclein aggregation, it may also dysregulate other key pathways, leading to reduced ATP levels and increased oxidative stress.

Given its role in key PD pathways, it is of major interest to see that sirtuin-2, in the proteomic study, was reduced across six affected PD regions, and validated in 3 regions. Importantly, sirtuin-2 is reduced in the frontal cortex (unaffected region) and temporal cortex (mildly affected region) of early stage PD brain. Interestingly, Sirtuin-2 is predominantly expressed in oligodendroglia, and is critical to their differentiation and myelination [81]. Here, we find SIRT2, ACAT1 and PYGL to be significantly enriched in oligodendrocyte cell-type specific proteomic data. Oligodendrocyte-specific proteins, such as the myelinating proteins MBP, MOG, MOBP, PLP, OMG, MPM2, and CNP, are also downregulated across disease stages, including in unaffected regions by > 1.5 fold compared to controls. This alteration in oligodendrocyte-specific proteins in ‘yet to be affected’ regions in PD brains, supports recent transcriptomic studies implicating oligodendrocyte dysfunction as an early and important event in PD [82–84]. The reduction in sirtuin-2 in early PD may therefore (i) be linked to oligodendrocyte dysfunction and represent an early mechanism by which mitochondrial metabolism and oxidative stress occurs, or (ii) may in some way be linked to a protective mechanism against, and prior to the emergence of, alpha-synuclein aggregation.

## Conclusions

**Fig. 9:**
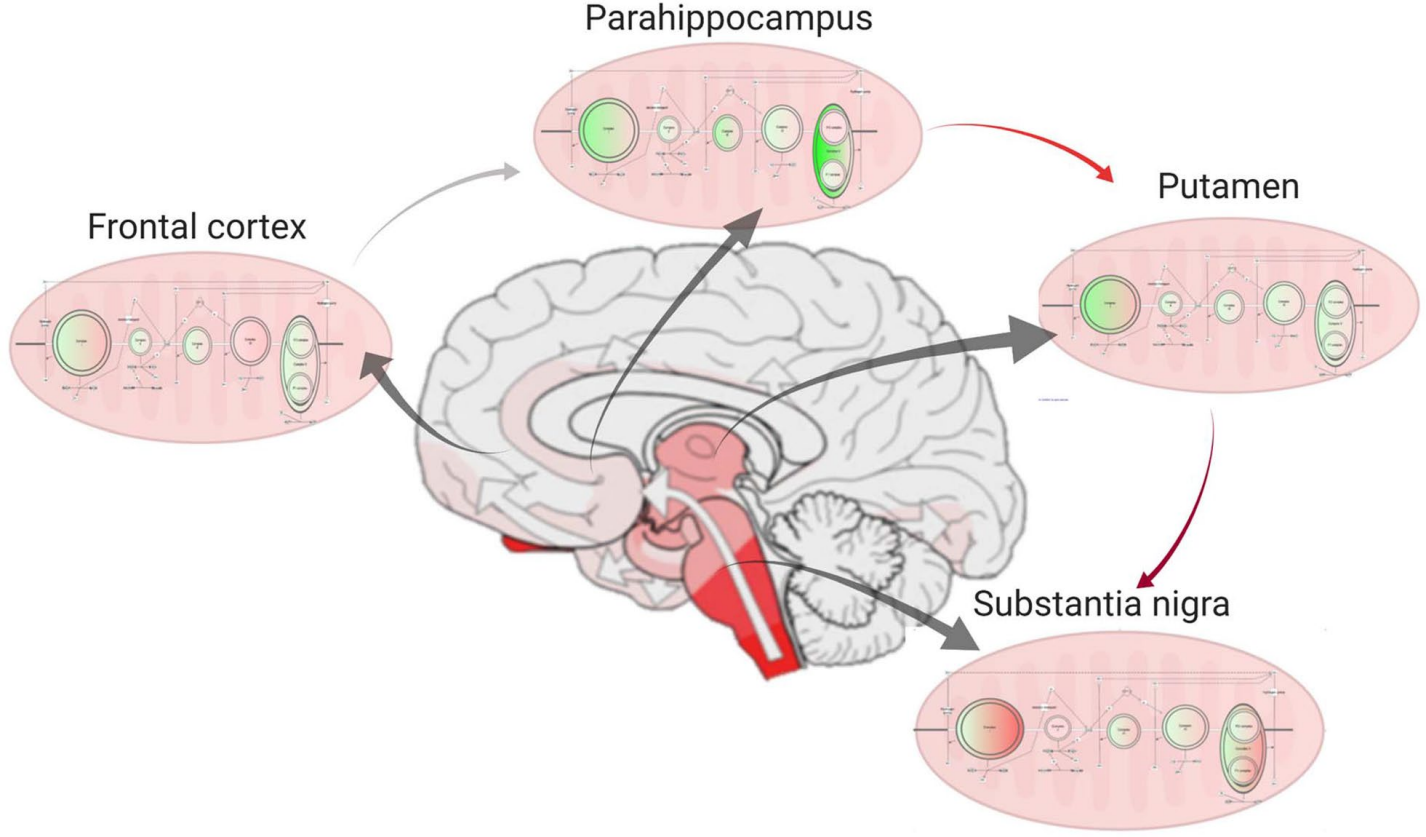
Progression of mitochondrial protein expression changes through the early PD brain. Schematic showing how mitochondrial proteins change in expression through the areas of the brain that are affected by alpha-synuclein pathology in early stage PD. Green represents downregulated proteins and red represents upregulated proteins when compared to controls. Arrows represent severity of the pathology across regions of the brain. Middle brain image represents how the pathology has spread through the brain at Braak stage 3/4 (adapted from [85]) Mitochondrial expression diagrams were made using IPA software. Figure made with biorender.com

In summary, we provide a detailed and comprehensive assessment of the human PD brain proteome across disease stages to capture the early and common signatures of cells vulnerable to PD pathology. Here, we demonstrate the presence of mitochondrial dysfunction throughout the PD brain, irrespective of the level of pathology (Fig. 9). Critically, we show that this dysfunction occurs prior to, or concomitantly with, the earliest stages of alpha-synuclein aggregation, and certainly prior to neuronal loss, implicating mitochondrial dysfunction as an early driver of the disease, and not just an end-stage phenomenon. Furthermore, we highlight two proteins, sirtuin-2 and metallothionein 2, that significantly change in vulnerable PD regions prior to the appearance of alpha-synuclein pathology, and thus require further investigation as to their pathologic or compensatory role in PD. Together, our use of comparative brain proteomics in carefully selected regions from early stage disease reveals the pathways driving the pathogenesis of sporadic PD. Importantly, our identification of these pathways, rather than those implicated later in the disease, may highlight novel targets for therapeutic intervention that could slow the underlying progression of PD.

## Supplementary Information

**Additional file 1: Figure S1.** GO terms altered in early PD compared to controls and mitochondrial gene expression patterns (a) Bar charts indicating number of Gene Ontology (GO) terms that are represented in the dataset for each region as determined by Webgestalt and DAVID databases. Top chart shows Biological processes, middle the molecular functions and lower the cellular components. The pie chart adjacent to each chart shows the number of GO terms that overlap or are uniquely represented in the dataset for a region of severe pathology (substantia nigra), mild pathology (parahippocampus) and a region unaffected at Braak stage 3/4 (frontal cortex) as determined by GOview. Colours indicate the level each region is affected at Braak stage 3/4 as determined in Figure 1b. Protein heatmaps from (b) first mass spectrometry run and (c) second mass spectrometry run from IPA showing the level of expression change per protein in the Mitochondrial Dysfunction pathway across each brain region in Braak stage 3/4 compared to controls. Red indicated upregulation and green indicated downregulation compared to controls. Intensity of colour shows level of expression change with deeper colour indicating higher up- or down- regulation.

**Additional file 2: Figure S2.** Candidates for validation workflow A pie chart showing the proportion of mitochondrial proteins out of the total proteins that were detected. The box outlines the method used to select candidate proteins for validation. The final pie chart shows the proportion of mitochondrial proteins within the list of proteins that were selected for validation.

**Additional file 3: Figure S3.** Citrate synthase protein levels across the brain. Graph highlighting how much citrate synthase protein was detected per disease group and brain region. Two-way ANOVA with Sidak’s multiple comparisons determined that citrate synthase levels across disease groups were non-significant (p = 0.1647) whilst those between regions were significant (p < 0.0001). Figure and analysis made with GraphPad Prism 9.02.

**Additional file 4:Table S1.** Peptide list for candidates. Peptide chosen for each protein that was validated with multiple reaction monitoring.

**Additional file 5:Table S2.** Biological processes represented across brain regions for early PD cases compared to controls. Shaded box reflects  GO term represented in that brain region.

**Additional file 6:Table S3.** Molecular functions represented across brain regions for early PD cases compared to controls. Shaded box reflects GO term represented in that brain region.

**Additional file 7:Table S4.** Cellular components represented across brain regions for early PD cases compared to controls Shaded box reflects GO term represented in that brain region.

**Additional file 8: Table S5.** Comparison of candidate proteins to cell type transcriptomic and proteomic data. Each protein that was compared to downloaded datasets from the articles listed to determine which cell type they associated with. * indicates < 5% FDR significant association in enrichment analysis.

**Additional File 9: Table S6.** Proteins with > 1.5 fold expression change within each region for early PD compared to controls. Protein ID is shown for each protein in the list. Red shading indicates upregulation and green shading indicates downregulation. Each region is highlighted to show the severity of pathology present in that region at Braak stage 3/4 with deepest severely affected > moderately affected > mildly affected > not affected.

**Additional file 10: Table S7.** List of GO terms represented between early and late PD cases in the frontal cortex. Biological processes, molecular functions and cellular components are split into different columns. Top 30 GO terms that overlap between both sets of brains compared to controls are given as well as those only found in early PD and only found in late PD.

**Additional file 11: Table S8.** List of GO terms represented between early and late PD cases in putamen. Biological processes, molecular functions and cellular components are split into different columns. Top 30 GO terms that overlap between both sets of brains compared to controls are given as well as those only found in early PD and only found in late PD.

## Data Availability

The datasets generated and/or analysed during the current study are available from the corresponding author on reasonable request.

## Abbreviations

PD: Parkinson’s disease
LB: Lewy bodies
LC-MS/MS: Liquid chromatography with tandem mass spectrometry
GO: Gene ontology
MRM: Multiple reaction monitoring
UPLC: Ultra performance liquid chromatography
PMD: Post-mortem delay
FFPE: Formalin-fixed paraffin-embedded
TBST: Tris buffered saline with tween
DAB: Diaminobenzidine
IMS: Industrial methylated spirit
IPA: Ingenuity pathway analysis
BP: Biological processes
MF: Molecular function
CC: Cellular component
HPLC-ESI-MS/MS: High performance liquid chromatography coupled with electrospray ionisation with tandem mass spectrometry
TCA: Tricarboxylic acid
PCA: Principle components analysis
ATP: Adenosine triphosphate
ADP: Adenosine diphosphate
NAD: Nicotinamide adenine dinucleotide
G6PD: Glucose-6-phosphate dehydrogenase
