# Supplementary figures and images for: Mitochondrial dysfunction is a key pathological driver of early stage Parkinson’s
Source: Acta Neuropathol Commun. 2022 Sep 8;10:134. doi: 10.1186/s40478-022-01424-6 (PMC9461181; doi:10.1186/s40478-022-01424-6)

## Slide 1
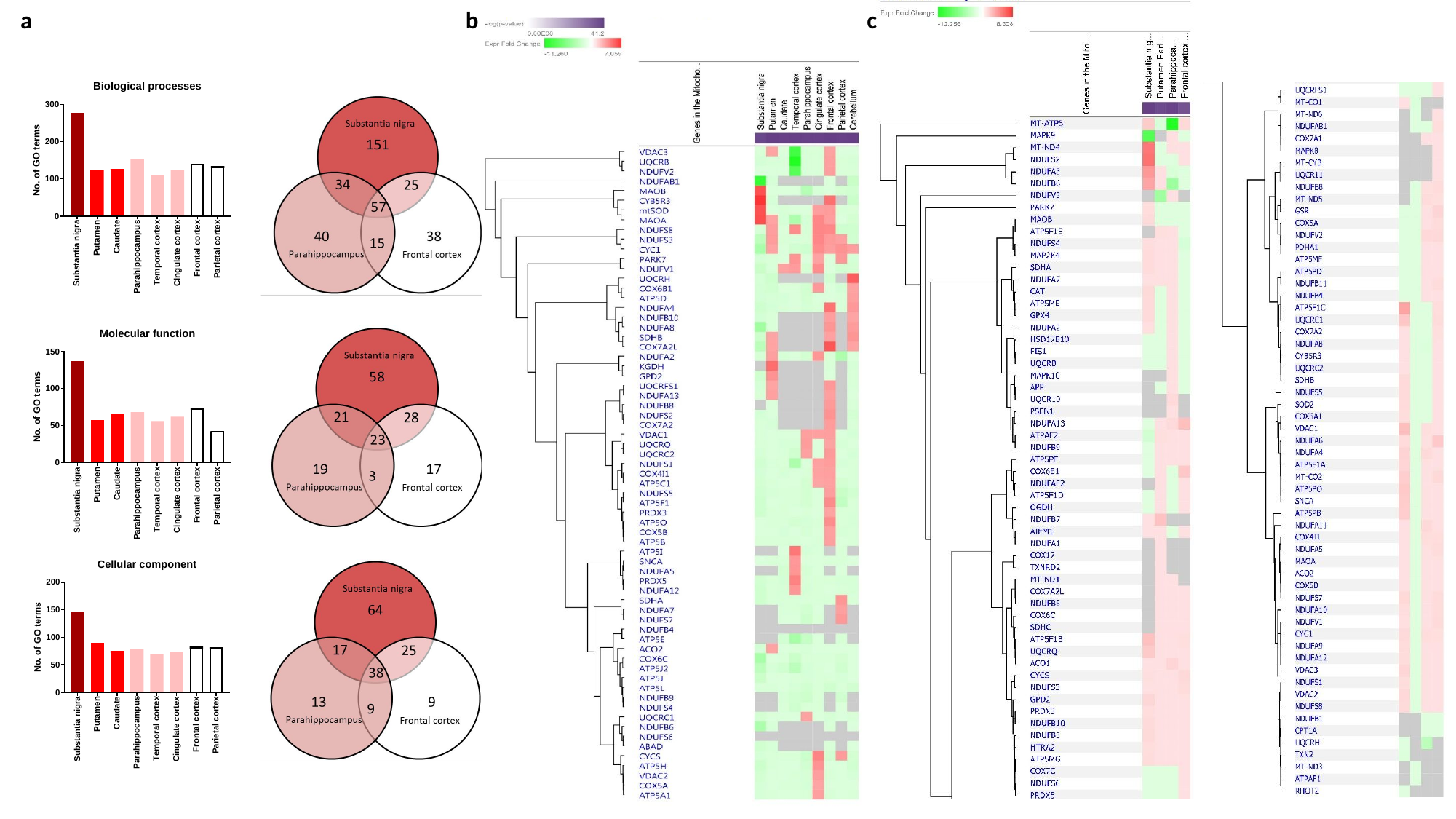

a
b
c

Supplement: Supplementary file 1 — Additional file 1: Figure S1. GO terms altered in early PD compared to controls and mitochondrial gene expression patterns (a) Bar charts indicating number of Gene Ontology (GO) terms that are represented in the dataset for each region as determined by Webgestalt and DAVID databases. Top chart shows Biological processes, middle the molecular functions and lower the cellular components. The pie chart adjacent to each chart shows the number of GO terms that overlap or are uniquely represented in the dataset for a region of severe pathology (substantia nigra), mild pathology (parahippocampus) and a region unaffected at Braak stage 3/4 (frontal cortex) as determined by GOview. Colours indicate the level each region is affected at Braak stage 3/4 as determined in Figure 1b. Protein heatmaps from (b) first mass spectrometry run and (c) second mass spectrometry run from IPA showing the level of expression change per protein in the Mitochondrial Dysfunction pathway across each brain region in Braak stage 3/4 compared to controls. Red indicated upregulation and green indicated downregulation compared to controls. Intensity of colour shows level of expression change with deeper colour indicating higher up- or down- regulation. [file 40478_2022_1424_MOESM1_ESM.pptx]

## Slide 1
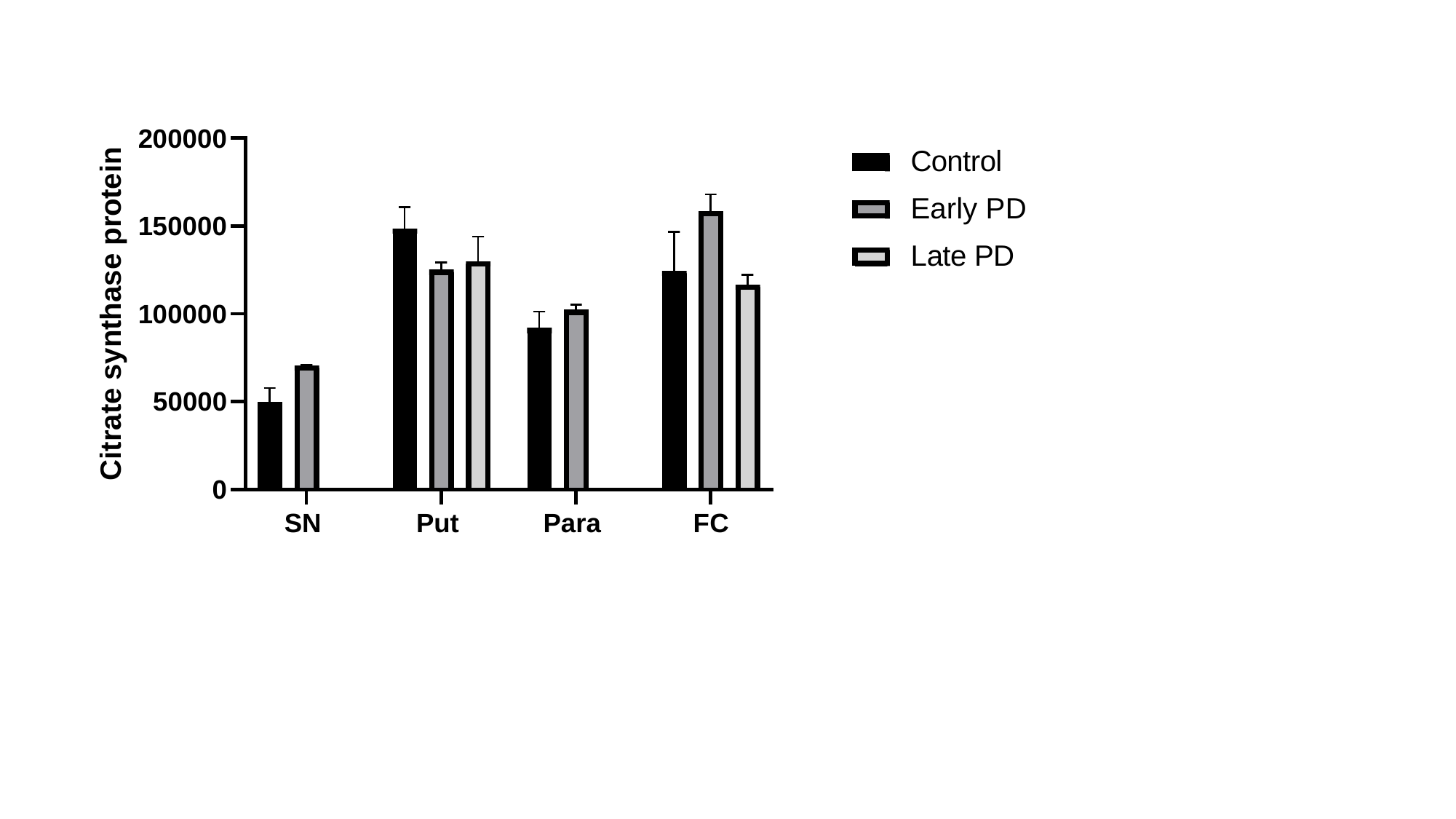

Supplement: Supplementary file 3 — Additional file 3: Figure S3. Citrate synthase protein levels across the brain. Graph highlighting how much citrate synthase protein was detected per disease group and brain region. Two-way ANOVA with Sidak’s multiple comparisons determined that citrate synthase levels across disease groups were non-significant (p = 0.1647) whilst those between regions were significant (p < 0.0001). Figure and analysis made with GraphPad Prism 9.02. [file 40478_2022_1424_MOESM3_ESM.pptx]
