# Supplementary material for: Mitochondrial dysfunction is a key pathological driver of early stage Parkinson’s
Source: Acta Neuropathol Commun. 2022 Sep 8;10:134. doi: 10.1186/s40478-022-01424-6 (PMC9461181; doi:10.1186/s40478-022-01424-6)
Supplement: Supplementary file 2 — Additional file 2: Figure S2. Candidates for validation workflow A pie chart showing the proportion of mitochondrial proteins out of the total proteins that were detected. The box outlines the method used to select candidate proteins for validation. The final pie chart shows the proportion of mitochondrial proteins within the list of proteins that were selected for validation. [file 40478_2022_1424_MOESM2_ESM.pptx]

## Slide 1
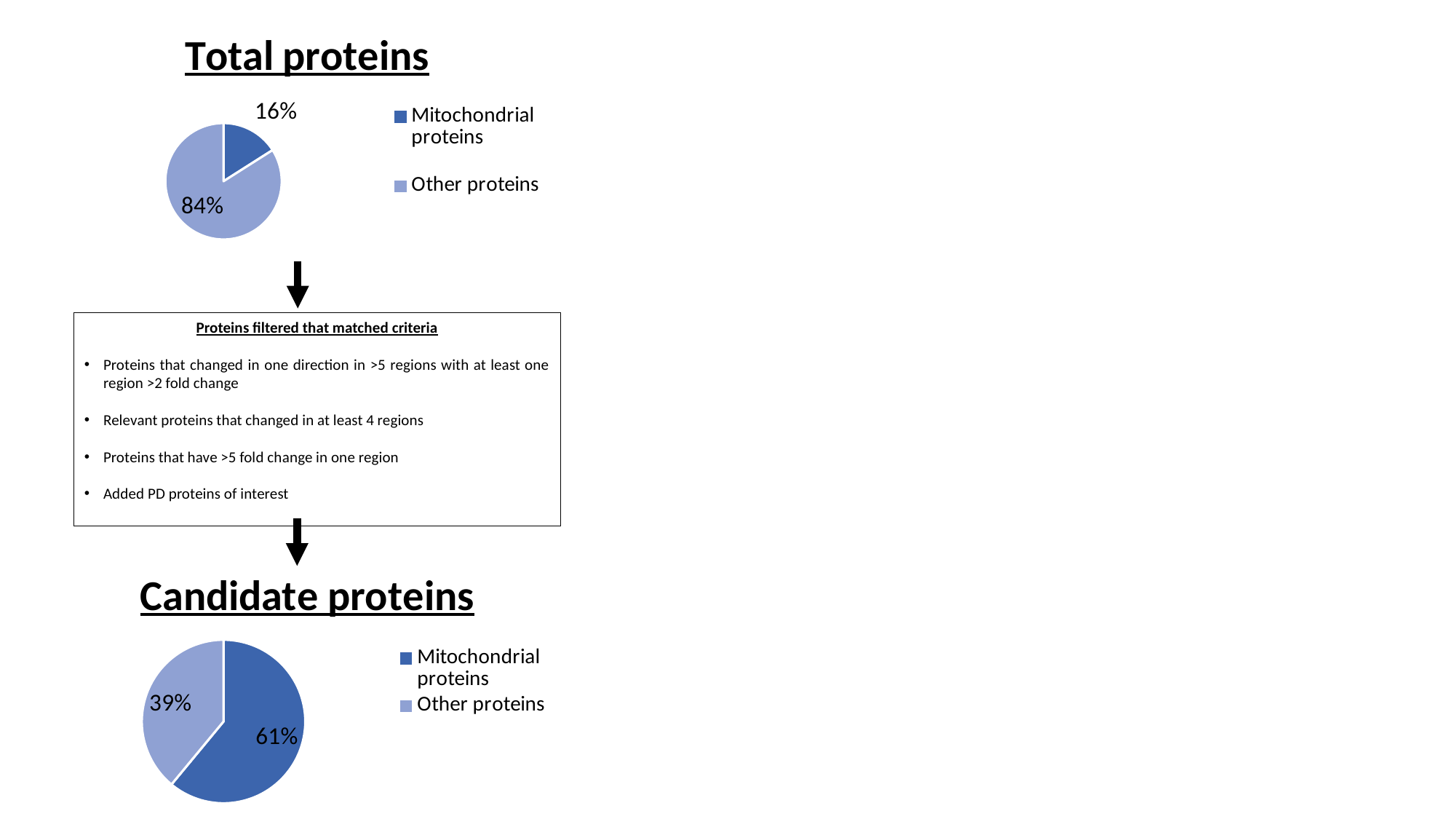

### Chart: Total proteins
| Category | |
|---|---|
| Mitochondrial proteins | 0.16 |
| Other proteins | 0.84 |Proteins filtered that matched criteria
Proteins that changed in one direction in >5 regions with at least one region >2 fold change
Relevant proteins that changed in at least 4 regions
Proteins that have >5 fold change in one region
Added PD proteins of interest
### Chart: Candidate proteins
| Category | |
|---|---|
| Mitochondrial proteins | 0.61 |
| Other proteins | 0.39 |
### Chart: Candidate proteins to validate
| Category |
|---|
